# Cancer Metabolism: The Role of ROS in DNA Damage and Induction of Apoptosis in Cancer Cells

**DOI:** 10.3390/metabo13070796

**Published:** 2023-06-27

**Authors:** Yongxia Zhao, Xiaochun Ye, Zhifeng Xiong, Awais Ihsan, Irma Ares, Marta Martínez, Bernardo Lopez-Torres, María-Rosa Martínez-Larrañaga, Arturo Anadón, Xu Wang, María-Aránzazu Martínez

**Affiliations:** 1 National Reference Laboratory of Veterinary Drug Residues (HZAU) and MAO Key Laboratory for Detection of Veterinary Drug Residues, Huazhong Agricultural University, Wuhan 430070, China; 2 Department of Animal Nutrition and Feed Science, Huazhong Agricultural University, Wuhan 430070, China; 3 Department of Biosciences, COMSATS University Islamabad, Sahiwal Campus, Sahiwal 57000, Pakistan; 4 Department of Pharmacology and Toxicology, Faculty of Veterinary Medicine, Universidad Complutense de Madrid (UCM), and Research Institute Hospital 12 de Octubre (i+12), 28040 Madrid, Spain

**Keywords:** cancer, ROS, DNA damage, apoptosis, oxidative stress, cancer metabolism

## Abstract

Cancer is a huge challenge for people worldwide. High reactive oxygen species (ROS) levels are a recognized hallmark of cancer and an important aspect of cancer treatment research. Abnormally elevated ROS levels are often attributable to alterations in cellular metabolic activities and increased oxidative stress, which affects both the development and maintenance of cancer. Moderately high levels of ROS are beneficial to maintain tumor cell genesis and development, while toxic levels of ROS have been shown to be an important force in destroying cancer cells. ROS has become an important anticancer target based on the proapoptotic effect of toxic levels of ROS. Therefore, this review summarizes the role of increased ROS in DNA damage and the apoptosis of cancer cells caused by changes in cancer cell metabolism, as well as various anticancer therapies targeting ROS generation, in order to provide references for cancer therapies based on ROS generation.

## 1. Introduction

In the 21st century, cancer has become one of the most important diseases threatening human life and health. Lung cancer is the most common cancer type and the leading cause of death in both male and female patients. In contrast, breast cancer is most common in women, accounting for 11.7% of all female cancer cases in 2020 [1,2]. In addition, the incidence of colorectal cancer in young adults is still rising [3,4,5]. Global colorectal cancer deaths are expected to increase by more than 60% by 2035 [6]. Cancer contributes greatly to the global mortality rate. In today’s world, cancer is frequently discussed. Where does the fear of cancer come from? Since cancer became the leading cause of morbidity and mortality worldwide, an increasing number of clinical cancer treatment strategies, such as surgery, chemotherapy drugs, radiotherapy and immunotherapy, and different combinations of these therapies, have been used to address the cancer crisis. However, these therapies have different limitations or produce different degrees of toxicity to the body, such as off-target effects, immunosuppression, and cancer cell resistance [7,8]. Therefore, it has forced us to find more effective anticancer strategies. Targeting ROS production would be a good approach for developing these strategies. At normal physiological concentrations, ROS are signal regulatory molecules for various physiological activities that play an important role in basic metabolism. However, when the concentrations of ROS in the body are not controlled, ROS plays a key role in the process of cancer. The abnormal increase in ROS level causes DNA damage, cancer cell metabolism changes, tumor metastasis, and tumor drug resistance, which are closely related to the occurrence and development of malignant cancer tumors [9,10,11].

Compared with normal cells, cancer cells show remarkable features, such as continuous proliferation, abnormal DNA damage response (DDR), and high levels of ROS [12]. The occurrence of cancer is related to a variety of factors, such as genetic factors, environmental and chemical pollution, bad living and eating habits, emotional abnormalities, and some endogenous factors. Research studies have shown that cancer cells can break through the limited cell proliferation limitation by expressing telomerase to lengthen telomere length and maintain unlimited proliferation ability even in the case of DNA damage [13]. Excessive DNA damage at the cellular level is associated with phenotypes such as cancer and premature aging [14]. Ataxia telangiectasia mutated (ATM) and ATM/Rad3-related (ATR) protein kinases are key regulators of the DDR pathway. DNA damage caused by high levels of ROS increases genomic instability and significantly increases the probability of ATM and ATR gene mutations, thereby increasing the risk of cancer [15,16,17]. Under oxidative stress, ROS-induced oxidative DNA damage has been reported to promote the development of colorectal cancer [18]. In addition, immune cells are the most important components of inflammation. Earlier studies have found that inflammation can accelerate the occurrence and development of cancer by affecting the metabolism-related processes of cells [19]. These unique characteristics of cancer cells have distinctive advantages for the development of cancer, which poses great challenges to clinical cancer treatment.

Compared with normal cells, cancer has the characteristics of cellular metabolic reprogramming, that is, cancer cells adapt metabolism to meet their own nutritional and material needs for continuous proliferation, thus promoting transformation and tumor development [20]. In recent years, it has been discovered that there are six major markers of cancer-related metabolic changes, mainly involving the interaction of nutrient uptake, biosynthesis, energy metabolism, and the tumor microenvironment [21]. Therefore, new metabolic markers of cancer are still emerging. Cancer cells adopt various metabolic adaptations to break through local limited oxygen and nutrient restrictions and maintain cancer cell growth and proliferation [22]. In 1924, German biochemist Otto Warburg found that, compared to cells in normal tissues, cancer cells tend to produce lactic acid via glucose fermentation even under conditions that provide sufficient oxygen [23], and this metabolic feature of cancer cells was of great significance for maintaining cell proliferation [24]. The reprogramming of energy metabolism is generally recognized as a significant marker of cancer [25]. Abnormally activated growth and survival signals promote the metabolic reprogramming of cancer cells, thereby increasing nutrient acquisition and biosynthesis. Chang et al. found that glucose competition between tumor cells and T cells in the tumor microenvironment can directly regulate cancer progression [26]. ROS is a by-product of cellular oxidative metabolism, and the energy source of CD4 T cell differentiation is highly dependent on aerobic glycolysis, and the demand for energy metabolism increases during T cell activation [27]. Therefore, the local concentration of ROS increases rapidly in a short time, and the break of redox homeostasis will promote the occurrence and development of cancer. In addition, the metabolic changes of cancer cells affect other types of cells in the tumor microenvironment, which also contributes greatly to the biosynthesis and proliferation of cancer cells. Chen et al. used bioinformatics tools to explain the relationship between colon cancer metabolism and the tumor microenvironment from the perspective of the glycolysis–cholesterol synthesis axis [28]. Just as important, ROS types and levels in cancer cells differ significantly at different stages of tumor progression, which also affects tumor signal transduction and tumor progression [29]. At present, there have been many research studies on ROS and cancer that have revealed the abnormal accumulation of ROS and the role of ROS in the process of cancer occurrence and progression. Consequently, the metabolic adaptation of cancer cells is a necessary condition for the occurrence and maintenance of tumors.

All cancer cells have obviously high levels of ROS, but excessive accumulation of ROS can aggravate oxidative damage of the DNA of cells and induce the apoptosis of cancer cells. Under normal circumstances, an appropriate amount of ROS is one of the necessary conditions for maintaining cell signaling and redox homeostasis, but a further increase in ROS levels due to exogenous stimulation or metabolic changes in cancer cells is carcinogenic. High levels of ROS in cancer cells put cancer cells in a state of high oxidative stress and lead to the oxidative stress-induced damage of proteins, lipids, DNA, and mitochondria [30], among which DNA is the most sensitive target of ROS. High ROS levels drive DNA damage and genomic instability, contributing to cancer development. However, when ROS levels in cancer cells accumulate toxic effects on cells, they also show obvious anticancer effects. ROS has become an important anticancer target for clinical cancer therapy based on its dual mechanism of action. Recent studies have shown that when NCX4040 (a unique nitric oxide donor) is used to treat tumors, glutathione (GSH) is consumed and ROS is generated, which results in oxidative DNA damage and the destruction of tumor cells [31]. Oxidative stress and inflammation are closely related to cancer and apoptosis [32]. Subsequent research studies have found that NCX4040 can also induce a TNF-dependent pathway, further increasing oxidative stress through NF-κB-mediated immune responses, leading to increased apoptosis (mediated by NCX4040) [33]. In addition, Popovici et al. revealed that *Usnea barbata* extract in canola oil showed anticancer potential through ROS generation leading to DNA damage and autophagy [34]. Therefore, there is a relatively low or high threshold for ROS accumulation to promote or inhibit cancer. 

Over the past few decades, the relationship between oxidative stress, ROS, and cancer has been elucidated. Current research reviews on cancer mainly focus on the adaptive metabolism of cancer cells and cancer development, the abnormal metabolism of cancer cells and the shaping of the tumor immune microenvironment, and the relationship between metabolic phenotypes and cancer. However, the relationship between ROS accumulation caused by cancer metabolism and DNA damage and the apoptosis of cancer cells has not been systematically elaborated. Therefore, this review article aims to systematically summarize the dual mechanism of promoting and inhibiting cancer via ROS accumulation in cancer cells and clarify how ROS levels induce DNA damage and promote the apoptosis of cancer cells. In addition, the various cancer treatment strategies targeting ROS generation that have been used in recent years are also summarized to provide references for further research on drugs, specifically with respect to targeting ROS generation in cancer cells or anticancer therapies.

## 2. ROS Levels in Cancer Cells and Cancer Development

### 2.1. Mechanisms of ROS Generation in Cancer

Both exogenous environmental stimuli and endogenous factors can lead to ROS generation. When exposed to exogenous stimuli such as air pollutants, heavy metals, radiation, and anticancer drugs, ROS will abnormally accumulate in cells, leading to oxidative stress and gene mutations, which may induce the transformation of normal cells into cancer cells [35]. Air pollution has long been recognized as one of the common factors endangering human health as it induces high levels of ROS generation in cells and leads to oxidative stress, posing a cancer risk to humans [36]. Research studies have shown that oxidative stress in the ROS-mediated pathway may be a key mechanism for the biological effects of ambient fine particulate matter [37]. Niture et al. found that the use of a low-concentration of Cd (1–10 nM) increases ROS generation, cell proliferation, and cell steatosis while activating fibrogenic/oncogenic signaling in HepaRG and hepatocellular carcinoma (HCC) cells [38]. In the skin of pigmented mice, ultraviolet radiation amplifies ROS generation, potentially increasing the risk of malignant melanoma [39,40]. Clinically, the use of anticancer drugs can induce ROS accumulation either directly or indirectly. In research studies on the antitumor activity of the polyphenol agrimol B (Agr), it was found that Agr could increase the generation of ROS [41]. In addition, the organic arsenic compound Aa-Z2 induces ROS accumulation by targeting pyruvate dehydrogenase kinase 1 (PDK-1) to induce osteosarcoma apoptosis [42]. On the other hand, endogenous factors in the body can also lead to the excessive generation of ROS. Endoplasmic reticulum stress enhances ROS generation [43]. Furthermore, tumor microbiota can regulate immune response and local inflammation through ROS generation, thus promoting tumor progression [44]. In conclusion, ROS levels in the body and cells are affected by internal and external environmental factors.

**Figure 1:**
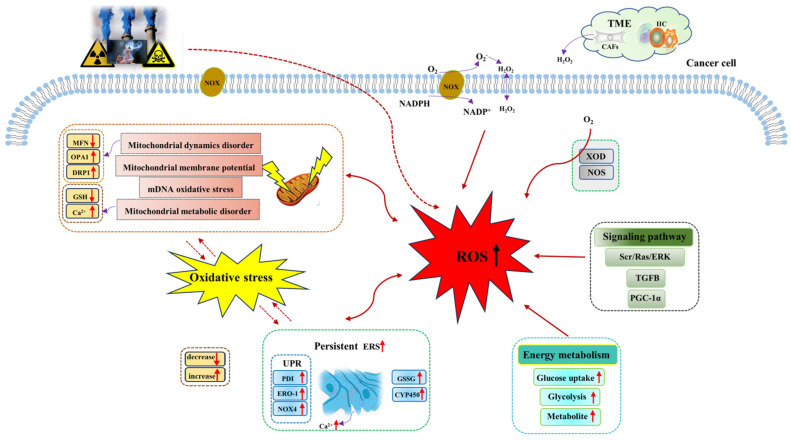
Mechanisms of ROS generation in cancer cells. Both exogenous stimuli and endogenous factors can induce the generation of ROS, which are mainly generated from mitochondria, NOX, endoplasmic reticulum, energy metabolism, and other pathways. Abbreviations: CYP450, cytochrome P450; CAFs, cancer-associated fibroblasts; DRP1, dynamin-related protein 1; ERK, extracellular signal -regulated kinase; ERO−1, endoplasmic reticulum oxidoreductin−1; ERS, endoplasmic reticulum stress; GSH, glutathione; GSSG, oxidized glutathione; H_2_O_2_, hydrogen peroxide; IIC, infiltrating immune cells; MFN, mitochondrial fusion protein; NADPH, nicotinamide adenine dinucleotide phosphate; NOX, NADPH oxidases; NOS, nitric oxide synthase; NOX4, NADPH oxidase 4; O_2_, oxygen; O_2_^−^, superoxide; OPA1, optic atrophy 1; PGC−1α, peroxisome proliferator-activated receptor-gamma-coactivator−1alpha; PDI, protein disulfide isomerase; Ras, guanine nucleotide-binding protein; ROS, reactive oxygen species; Src, non-receptor tyrosine kinase; TME, tumor microenvironment; TGFB, transforming growth factor beta; UPR, unfolding protein response; XOD, xanthine oxidase.

The metabolic reprogramming characteristic enables cancer cells to generate ROS at higher levels than normal cells through a variety of biological pathways. In tumor tissues, the rapid energy metabolism and proliferation of tumor cells generate a large number of ROS, including superoxide (O_2_^−^), hydroxyl radical (−OH), hydrogen peroxide (H_2_O_2_), and lipid hydroperoxide (LOOH). ROS mainly comes from mitochondria [45]. Mitochondria provide energy for various biological processes in cancer cells, and ROS is produced from oxidative phosphorylation (OXPHOS). However, research studies have shown that elevated ROS levels will induce mitochondrial DNA (mtDNA) damage and mitochondrial dysfunction, which will further aggravate ROS generation, resulting in a vicious cycle of ROS generation and mtDNA damage [46]. In addition, the generation of ROS also depends on the regulation of NADPH oxidases (NOXs), nitric oxide synthase (NOS), and xanthine oxidase. Among them, NOXs are the main regulatory enzymes of ROS regulation [47]. Research studies have shown that NOX1 expression significantly increases the regulation of O_2_^−^ and maintains the proliferative phenotype of colon cancer cells [48,49]. In addition, there are both tumor cells and a variety of normal cells in the tumor microenvironment, in which stromal cell cancer-associated fibroblast-derived H_2_O_2_ leads to extracellular oxidative stress and promotes tumor growth and invasion [50]. Therefore, ROS accumulation in cancer cells is mediated by multiple biological pathways (Figure 1).

Sources of ROS in cancer cells mainly include mitochondria, NOX, and endoplasmic reticulum [51]. ROS is a natural by-product of cellular aerobic metabolism and plays a significant role in intercellular signaling pathways, redox homeostasis maintenance, and body metabolism. Cancer cells have significantly higher levels of oxidative stress, and they can maintain high ROS levels through various mechanisms to support the development of cancer. Mitochondrial dysfunction and metabolic alterations are known to be the main sources of ROS [52]. Mitochondria are highly dynamic organelles, and their ability to regulate exogenous or endogenous stimuli depends on the close relationship between mitochondrial dynamics and mitochondrial energy metabolism. In the cell, mitochondrial dynamics play a role in regulating mitochondrial fusion and division and the balance between them. When oxidative stress or cell metabolism and other factors cause disorders affecting mitochondrial dynamics (such as the imbalance of the mitochondrial fusion and division), mitochondrial function will become increasingly abnormal, thus inducing an increase in ROS generated by mitochondria and enhancing tumor invasion [53]. In studying the specific mechanism of ouabain (a steroid hormone) against glioblastoma, Yan et al. found that ouabain induced mitochondrial damage and led to increased ROS generation through the phosphorylation of p66Shc (isoform derived from Shc1 gene), mediated by the Src/Ras/ERK signaling pathway [54]. NOX induces the intracellular accumulation of ROS. Research studies have shown that NOX is one of the sources of ROS in melanoma, playing a crucial role in all aspects of melanoma development [55,56]. Brevilin A (Brv-A), a sesquiterpene lactone compound of *Centipeda minima* induces ROS generation through NOX2 and NOX3 regulation [57]. Baldi et al. showed that the ferroptosis-related gene-activated TGFB pathway could stimulate ROS generation to promote tumor invasion and progression [58]. In addition, Biswas et al. found that MAO-A-mediated ROS accumulation is a key factor in cancer apoptosis [59]. All these facts suggest that the abnormal accumulation of ROS in cancer cells is mediated by multiple mechanisms for tumor response. Table 1 lists the main factors of ROS generation. 

ROS is known to play a significant role in the development and maintenance of malignant tumors. ROS levels in cancer cells are dynamic due to complex changes in the exogenous environment and changes in the endogenous metabolism of the body, which are regulated by various metabolic ways. The metabolic reprogramming of cancer cells is a prominent feature of cancer that makes ROS generation more favorable. Therefore, it is necessary to study the correlation between ROS levels and cancer metabolism further.

### 2.2. The Dual Role of ROS in Promoting and Suppressing Cancer Cells

The abnormal metabolic behavior of cancer cells is to support the need for cancer cells to maintain their development in harsh environments such as lack of oxygen and nutrition [22]. Because cancer cells are constantly meeting their increased energy and biosynthesis needs, they must “broaden” their metabolic pathways autonomously. All cells, including cancer cells, require glucose metabolism for energy, and glucose metabolism is closely related to redox homeostasis. However, the energy metabolic pathway of cancer cells is classic aerobic glycolysis, even when oxygen is plentiful. The rapid uptake of glucose is accompanied by an increased rate of glycolysis. In precursor B-cell acute lymphoblastic leukemia cells, the glucose analog 1,5-anhydroglucitol interrupts mitochondrial respiration by up-regulating pyruvate dehydrogenase kinase 4 (PDK4), thereby increasing the rate of glycolysis, and enhanced glycolysis leads to oxidative stress in tumor cells by increasing ROS levels [76,77]. Additionally, the decreased O_2_^−^ level under hypoxia conditions inhibits the AMP-activated protein kinase (AMPK) activation and shifts metabolism from glycolysis to oxidative phosphorylation [78]. Overall, there is a close relationship between ROS homeostasis and the regulation of metabolism and cancer development.

An imbalance between redox homeostasis and oxidative stress will inevitably result from the accumulation of abundant metabolites from the hyperactive metabolic activities of cancer cells. Continuous oxidative stress will lead to a large accumulation of ROS within cancer cells, and the effect of ROS on cancer progression has been shown to be paradoxical [79,80,81]. High levels of ROS that do not cause cell death can promote cancer cell growth, metastasis, and angiogenesis. However, when ROS generation reaches a level that cancer cells cannot withstand (toxic level), it will kill cancer cells, that is, it has an antitumor effect. Therefore, the amount of ROS accumulation affects both the development and maintenance of cancer. The level of ROS in cancer cells is a double-edged sword because, as of now, no clear value that defines whether levels of oxidative stress are low or high has been established. The ROS generation mechanism in cancer cells is complex and key to maintaining redox homeostasis.

#### 2.2.1. High Levels of Steady-State ROS Play a Role in Promoting Cancer

The moderate accumulation of ROS is conducive to tumor maintenance and development [80]. Increased ROS generation or decreased ROS clearance due to exogenous or endogenous factors will put cells in a state of oxidative stress [82]. Oxidative stress can cause oxidative damage to biological macromolecules, among which DNA oxidative damage can cause mutation accumulation and genome instability, which is a favorable inducing factor for tumorigenesis. Oxidative DNA damage includes base oxidation, deoxyribose oxidation, single-strand breaks (SSBs), and DNA double-strand breaks (DSBs) [83]. Among the four bases, guanine is the most easily oxidized DNA base because it has the lowest redox potential [84], and its extensive accumulation of oxidation product 8-oxoguanine (8-oxoG) is closely related to tumorigenesis [85]. The base excision repair pathway (BER) is a key repair mechanism for oxidative DNA damage. When the important base excision repair mechanism is inhibited or cannot repair DNA damage in time, it will lead to increased gene mutations and greatly increase the probability of tumor induction [86]. Equally important, due to the strong adaptive capacity of cancer cells, cancer cells will respond to moderately high levels of ROS and further enhance the antioxidant system by inducing an increase in antioxidant enzymes and antioxidant proteins, thus continuing to maintain the development of cancer. Therefore, moderately elevated ROS is beneficial for cancer transformation.

**Figure 2:**
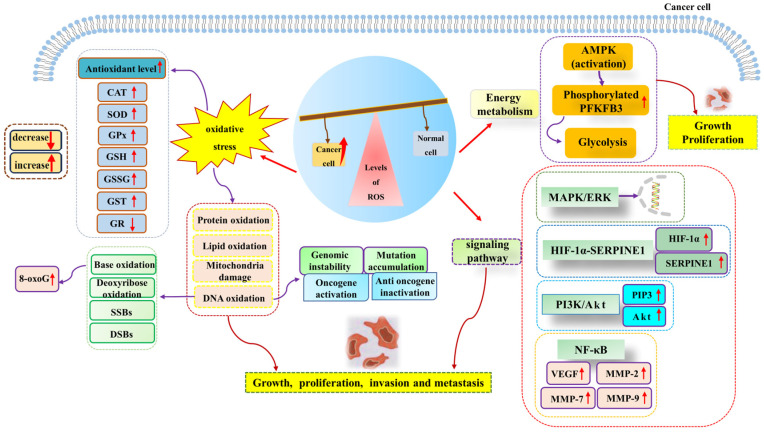
High levels of ROS promote cancer cell growth, proliferation, invasion, and metastasis. ROS generation can induce oxidative stress, energy metabolism, cancer-promoting signal pathways, and other pathways to promote the occurrence and development of cancer. Abbreviations: AMPK, adenosine 5′-monophosphate (AMP)-activated protein kinase; Akt, protein kinase B; CAT, catalase; DSBs, DNA double-strand breaks; ERK, extracellular regulated protein kinase; GR, glutathione reductase; GPX, glutathione peroxidase; GSH, glutathione; GSSG, oxidized glutathione; GST, glutathione-S-transferase; HIF-1α, hypoxia-inducible factor-1α; MAPK, mitogen-activated protein kinase; MMP-2, matrix metalloproteinase-2; MMP-7, matrix metalloproteinase-7; MMP-9, matrix metalloproteinase-9; NF-κB, nuclear factor kappa-B; PI3K, phosphatidyl-inositol-3-kinase; PIP3, phosphatidyl-inositol 3,4,5-trisphosphate; PFKFB3, 6-phosphofructo-2-kinase/fructose-2,6-bisphosphatase; ROS, reactive oxygen species; SOD, superoxide dismutase; SERPINE1, serine protease inhibitor family E member 1; SSBs, single-strand breaks; VEGF, vascular endothelial growth factor; 8-oxoG, 8-oxoguanine.

Many research studies have shown that excessive ROS generation has a pro-cancer effect [79,87]. High levels of ROS are associated with the development and progression of many malignancies. Adaptation to hypoxia, metabolic rate, oncogenic mutations, and the activation of pro-tumor signaling can all be inductors of tumorigenesis. It has been reported that hypoxia-induced ROS up-regulates the expression of matrix metalloproteinase-2 and matrix metalloproteinase-9 through the NF-κB signaling pathway, thus playing a role in promoting aneurysms [88]. Hypoxia-induced ROS can also enhance the proliferation, migration, and invasion of glioblastoma by driving the HIF-1α-SERPINE1 signaling pathway, thus exacerbating tumor progression [89]. In addition, Gao et al. suggested that the sterol-regulatory element binding protein 1 (SREBP1) promotes colon cancer cell invasion via the NF-κB–MMP7 axis by increasing ROS [90]. O_2_^−^ acts as a positive regulator of the Rac1-PI3K signaling pathway, which is associated with stimulating the migration and invasion of Nex10C mouse melanoma cells [91]. On the other hand, high levels of ROS homeostasis and glycolysis are hallmark features of cancer. In leukemia, ROS over generation affects glycolysis. It promotes the proliferation of acute myeloid leukemia by up-regulating the expression of 6-phosphofructo-2-kinase/fructose-2,6-bisphosphatase (PFKFB3), a key glycolytic regulator [92]. Furthermore, elevated glycolysis levels can promote the progression of precursor B-cell acute lymphocytic leukemia (pre-B AML) by activating MAPK/ERK pathways by increasing intracellular ROS levels [77]. The above research studies have shown that increased ROS accumulation can promote tumor progression through a variety of signaling pathways (Figure 2).

#### 2.2.2. Toxic Levels of ROS Play a Pro-Apoptotic Role

Since ROS levels play a role in promoting cancer, a further increase in ROS levels can promote antitumor signaling and trigger oxidative stress, thus inducing tumor cell apoptosis through the pro-apoptotic pathway. The imbalance of ROS induces adverse cancer outcomes by affecting signal transduction within the DDR [93] because the accumulation of ROS can be toxic to cells, and toxic levels of ROS have been shown to affect replication and transcription by inducing DNA strand breaks and base oxidation, which may lead to the accumulation of mutations in organisms and the transformation of tumors [94]. It was found that due to the increased expression of the tumor-suppressant gene deleted in liver cancer 1 (DLC1), ROS could further inhibit the DYRK1A–EGFR axis, triggering DNA damage, while the DNA double-strand break marker protein γH2AX was up-regulated and the DNA repair-related proteins p-BRCA1 and RAD51 were down-regulated, which contributed to tumor inhibition [95]. Irreversible DNA damage is a key factor in tumor cell apoptosis. For example, through ROS inducers such as chemotherapy, severe DNA damage causes irreparable damage to cells and eventually leads to tumor cell death [80]. Moreover, ROS can not only cause oxidative DNA damage and directly drive the apoptosis of lung cancer cells, but also cause endoplasmic reticulum stress in cancer cells to promote the translocation of calreticulin (multifunctional soluble protein) to the cell membrane, resulting in DNA damage and the expression and release of nuclear high mobility group box (HMGB1) and heat shock proteins (HSP90), thus inducing immunogenic cell death [96]. In addition, research studies have shown that metal-based anticancer chemotherapeutic agents can cause lethal damage to malignant tumors by inducing cytotoxic effects caused by multiple modes, including ROS-induced DNA damage and angiogenesis [97]. Therefore, DNA damage is a key ROS-mediated apoptosis signaling pathway in tumor cells.

Apoptosis is an important homeostatic mechanism of the body that plays an important role in maintaining the homeostasis of normal cells. Cancerous cells can break through the age limit and have the ability to continue to proliferate, which is also the reason for the occurrence of tumors. The apoptosis of cancer cells allows for the development of tumors to be controlled, which also brings greater hope to cancer treatment. At present, there are three major apoptotic pathways: (i) the endogenous mitochondrial apoptosis signaling pathway, (ii) the death receptor pathway, and (iii) the endoplasmic reticulum signaling pathway. It has been demonstrated that ROS generated in cancer cells induces apoptosis through the activation of these apoptotic signaling pathways and plays a role in tumor inhibition [98,99].

Toxic levels of ROS can activate members of the mitogen-activated protein kinase (MAPK) family, among which the activation of the c-Jun N-terminal kinase (JNK) signaling pathway (one of the major signaling cassettes of MAPK signaling pathway) can lead to intrinsic or extrinsic apoptotic signaling to initiate apoptosis [79,100]. Research studies have shown that JNK phosphorylation plays a role in inducing the apoptosis of human hepatocellular carcinoma BEL-7402 and HepG2 cells, specifically through the ROS-JNK-P53 signaling pathway [101]. In addition, the activated JNK signaling pathway is a key link in the apoptosis of intrahepatic cholangiocarcinoma cells by MLN2238 [102]. Furthermore, the ROS-mediated PI3K/Akt/mTOR and MAPK signaling pathways are the mechanisms by which CBL0137 promotes ROS generation and induces the apoptosis and autophagy of aggressive B-cell non-Hodgkin’s lymphoma cells [103]. Therefore, the occurrence of cancer cell apoptosis largely depends on the JNK signaling pathway.

Among the many apoptosis signaling pathways, the mitochondrial apoptosis signaling pathway plays an irreplaceable role in cancer cell apoptosis. Mitochondria are not only the “energy factories” of cells, but also the regulatory centers of cell survival and death. Mitochondrial dysfunction leads to excessive ROS generation, which leads to oxidative stress and aggravates mitochondrial dysfunction, leading to apoptosis. In addition, when cytokines, death receptors, cellular metabolites, and other factors stimulate mitochondria and affect the mitochondrial electron transport chain and energy metabolism, it is likely to activate the endogenous mitochondrial apoptosis signaling pathway, leading to cell apoptosis. Caspases play a special role in the mitochondrial intrinsic apoptosis signaling pathway and death receptor apoptosis signaling pathway, and its activation is one of the signs of cell apoptosis. ROS causes mitochondrial membrane damage and releases cytochrome c into the cytoplasm, which forms a complex with apoptotic protein activating factor 1 (Apaf-1) and pro-caspase-9 to induce the cleavage of caspase-3 and caspase-7, eventually leading to DNA fragmentation and cell death [80,104]. In the death receptor pathway, the ligand binds to the death receptor and causes the activation of caspase-3, which leads to the cleavage of downstream caspase 3 and the cleavage of Bid (Bcl-2 family protein) to Bid, then truncated Bid is transported to the mitochondria to mediate the release and transport of cytochrome c [105]. In addition, the excessive accumulation of ROS can also act by affecting the activity of apoptosis-related factors such as Bcl-2 family proteins. Bcl-2 family members also regulate the mitochondrial apoptosis pathway through protein–protein interactions [106]. Any pro-apoptotic factor or signal can disrupt mitochondrial membrane potential by reducing the Bcl-2/Bax ratio in cancer cells, causing the mitochondria to release cytochrome c into the cytoplasm [57] and ultimately mediating apoptosis through the caspase-dependent death pathway.

**Figure 3:**
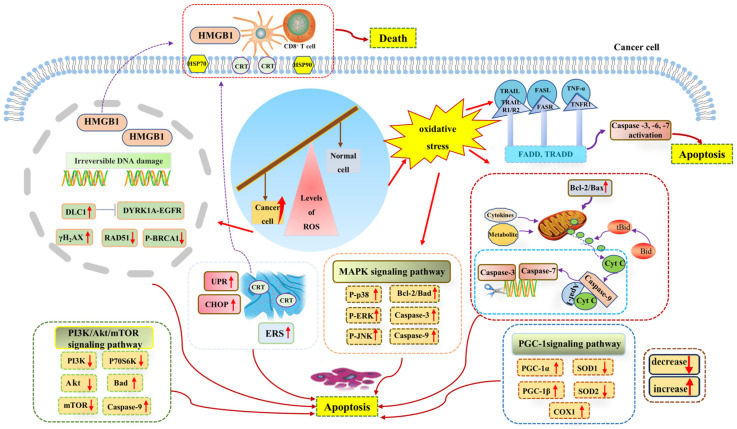
Levels of toxicity in the ROS-induced apoptosis of cancer cells. Toxic levels of ROS can cause irreversible DNA damage and promote a variety of apoptosis signaling pathways, such as the mitochondrial apoptosis signaling pathway, endoplasmic reticulum apoptosis signaling pathway, and a variety of pro-apoptotic signaling pathways, thus inducing cancer cell apoptosis. Abbreviations: Akt, protein kinase B; Apaf-1, apoptotic protease activating factor 1; Bad, pro-apoptotic protein; Bcl-2, B-cell lymphoma-2; Bax, BCL2-associated X-protein; Bid, BH3-interacting domain death agonist; CD8^+^ T cell, cytotoxic T lymphocytes; Cyt C, cytochrome C; CRT, calreticulin; Caspase-3, cysteinyl aspartate specific proteinase 3; Caspase-6, cysteinyl aspartate specific proteinase 6; Caspase-7, cysteinyl aspartate specific proteinase 7; Caspase-9, cysteinyl aspartate specific proteinase 9; CHOP, C/EBP-homologous protein; COX1, cyclooxygenase-1; DLC1, deleted in liver cancer-1 gene; DYRK1A, dual specificity tyrosine phosphorylation kinase 1a; EGFR, epidermal growth factor receptor; ERS, endoplasmic reticulum stress; FADD, Fas-associated protein with death domain; HMGB1, high mobility group box; HSP70, heat shock proteins 70; HSP90, heat shock proteins 90; MAPK, mitogen activated protein kinase; mTOR, mammalian target of rapamycin; P-p38, phosphorylated P38 protein; P-ERK, phosphorylated extracellular regulated protein kinases; P-JNK, phosphorylated c-Jun N-terminal kinase; P-BRCA1, phosphorylated breast cancer susceptibility genes; PI3K, phosphoinositide 3-kinase; P70S6K, p70 ribosomal protein kinase S6; PGC-1α, peroxisome proliferator-activated receptor-gamma-coactivator-1alpha; PGC-1β, peroxisome proliferator-activated receptor-gamma-coactivator-1beta; RAD51, RAD51 family of genes; ROS, reactive oxygen species; SOD1, superoxide dismutase-1; SOD2, superoxide dismutase-2; tBid, truncated Bid; TNF-α, tumor necrosis factor alpha; TNFR1, tumor necrosis factor receptor 1; TRAIL, tumor necrosis factor-related apoptosis-inducing ligand; TRADD, tumor necrosis factor receptor 1-associated death domain; UPR, unfolded protein response; γH_2_AX, phosphorylated histone H_2_AX.

It has been confirmed that ROS-induced apoptosis is related to endoplasmic reticulum stress [107,108]. The abnormal accumulation of ROS can induce endoplasmic reticulum stress-mediated apoptosis in various malignancies [57]. The endoplasmic reticulum is the site of protein synthesis. High levels of ROS accumulation can lead to endoplasmic reticulum stress and increase protein folding errors in the endoplasmic reticulum lumen, thereby amplifying unfolding protein response (UPR) signaling and inducing apoptosis [109,110]. Research studies have shown that C/EBP-homologous protein (CHOP) is highly expressed under endoplasmic reticulum stress and has a pro-apoptotic effect [111]. ROS-induced endoplasmic reticulum stress plays an important role in the apoptosis of gastric cancer cells [112]. Similarly, ROS levels regulate endoplasmic reticulum stress and mitochondrial dysfunction in oral cancer to induce PERK/eIF2α/CHOP signaling pathway activation and inhibit cell growth and apoptosis [113]. Several members of the TNF receptor family are capable of inducing apoptosis because of intracytoplasmic (death) domains that bind proteins in the cell death pathway. These include receptors for TNF-α, the FAS ligand (FASL), and the TNF-related apoptosis-inducing ligand or TRAIL. At least two receptors have been described for TRAIL: R1 and R2. R1 does not interact with the common adaptor molecule FAS-associated death domain (FADD), which binds to TNFR1 and FAS. The second receptor, R2, was also found to be widely distributed on tissues and, in contrast to R1, induced apoptosis via mechanisms that involved interactions with FADD [114]. It can be concluded that ROS-induced endoplasmic reticulum stress is also an important pathway of cancer cell apoptosis (Figure 3).

### 2.3. Changes in the Adaptation of Cancer Cells to ROS

Cancer cells employ multiple adaptive mechanisms in response to elevated ROS and oxidative stress [115]. In order to maintain genome stability under oxidative damage, cells have evolved a variety of DNA repair methods, such as BER, nucleotide excision repair, and mismatch repair. Experiments have shown that mice lacking the BER enzyme have a higher mutation rate and tumor incidence [116,117], and 8-oxoguanine DNA glycosylase1 (OGG1) initiates BER to remove 8-oxoG to avoid the accumulation of 8-oxoG and induce mutations [85]. In addition, mitochondrial homeostasis, antioxidant response, and hypoxia adaptation are also regulatory pathways in cancer cells when they are under ROS stress.

As we all know, endogenous ROS mainly comes from mitochondria and is a by-product of the oxidative phosphorylation of mitochondria. Research studies have demonstrated that uncontrolled mitochondrial ROS (mROS) affects normal cell survival and carcinogenic transformation [118]. However, there is also growing evidence to suggest that increased and sustained ROS leads to severely elevated oxidative stress, which leads to the accumulation of cellular damage at DNA, RNA, and protein levels, ultimately leading to cancer cell death. In order to maintain their survival, cancer cells are able to resist oxidative stress through a mitochondrial oxidative stress checkpoint mechanism, which is involved in metabolic regulation. DNA-PKcs (a multi-faceted player in DNA damage response) is not only a key regulator of DNA double-strand break repair but also a direct regulator of mitochondrial homeostasis. DNA-PKcs can directly interact with mitochondrial proteins ANT2 and VDAC2. The resulting DNA-PKcs/ANT2/VDAC2 (DAV complex) regulates mitochondrial ADP–ATP exchange and OXPHOS, thereby accelerating cellular oxidative stress repair [119].

The maintenance of redox homeostasis in cancer cells under high ROS levels supports cancer cell survival and proliferation. Cancer cells can make adaptive changes to oxidative stress, such as metabolic reprogramming, enhanced antioxidant capacity, and resistance to apoptosis, to meet the needs of their own continuous growth. As tumor-initiating cells, cancer stem-like cells (CSCs) have a more powerful antioxidant defense system to counteract, clear, and maintain tumorigenesis [120]. Research studies have shown that in order to adapt to high ROS levels during tumor progression, tumor cells survive high ROS levels through the high expression of antioxidant genes, increased GSH synthesis, and increased NADPH generation (through activation of AMPK, the pentose phosphate pathway, and reduced glutamine and folate metabolism) [121].

The antioxidant response is one of the key cellular defense mechanisms used by cancer cells to prevent oxidative stress, and nuclear factor erythroid 2-related factor 2 (Nrf2) is the core of the antioxidant reaction [122]. Nrf2 activation can not only participate in cancer metabolic reprogramming by promoting the high expression of metabolic enzymes but also maintain cellular redox homeostasis under oxidative stress. As one of the most important proteins involved in the control of the antioxidant response, Nrf2 has the ability to maintain cell homeostasis and support the metabolic process to cope with oxidative stress caused by abnormal accumulation of ROS. Nrf2 overactivation can up-regulate the expression of antioxidant enzymes, thereby increasing GSH synthesis to balance the high level of ROS accumulation. Research studies have shown that melanoma redox homeostasis mainly depends on Nrf2 to regulate the expression of antioxidant enzymes such as HO-1, SOD, CAT, and GPX [123]. In addition, the abnormal activation of the Nrf2/Keap1 (Kelch-like ECH-associated protein 1) signaling pathway is crucial to maintain cell homeostasis. The mechanism of its action is that oxidative stress leads to the release of Nrf2, which was originally coupled with the inhibitor Keap1. Nrf2 is then translocated to the nucleus to regulate the up-regulation of downstream antioxidant enzymes, thus playing a role in oxidative stress defense [124,125]. At the same time, the decrease in Nrf2 activity or its downstream targets can inhibit the apoptosis of cancer cells by mediating various intracellular signaling pathways to avoid their death [126,127]. Overall, these results indicate that cancer cells can both balance ROS by increasing the expression of antioxidant proteins and maintain their survival by resisting the apoptosis of cancer cells.

Cancer cells adapt to hypoxia through changes in energy metabolism. The maintenance of the continuous high proliferation state of cancer cells requires the consumption of a large amount of oxygen, so the hypoxic environment is considered to be a sign of the tumor microenvironment [128]. Hypoxic conditions lead to increased mROS generation, and long-term high ROS levels will cause oxidative damage to cells. Cancer cells adapt to proliferation and migration under hypoxic conditions through energy metabolism [89]. The Warburg effect (oncology) has pointed out that cancer cells have a different way of metabolizing energy compared to normal cells, namely, via aerobic glycolysis [23]. It is currently believed that glycolysis plays a role in the redox homeostasis of cancer. The intermediates produced by glycolysis enter other metabolic pathways to produce reducing agents, such as GSH, to maintain cell homeostasis [80]. In addition, cellular hypoxia adaptation depends on hypoxia-inducible factor-1α (HIF-1α) signaling, and ROS promotes the stability and activation of HIF-1α [129,130]. Thus, hypoxic adaptation also favors the survival of cancer cells under high ROS levels.

The occurrence and progression of cancer are closely related to bidirectional regulatory mechanisms below or above the threshold ROS level. Cancer cells also have the sufficient adaptive capacity to cope with the high level of ROS pressure and can maintain their survival and development through a variety of adaptive mechanisms, which is a favorable obstacle to tumor treatment.

## 3. Cancer Therapeutic Strategies Targeting ROS Generation

Recently, cancer treatment strategies that target ROS regulation in cancer cells have attracted increasing attention. The occurrence of cancer is a serious threat to human health. The current cancer treatment methods include radiotherapy, chemotherapy, surgery, immunotherapy, and molecular targeted therapy, and their cancer treatment effects have also been recognized in the clinical treatment of cancer patients. However, due to non-specific targeting, various adverse reactions, and the continuous emergence of tumor drug resistance, the application of traditional anticancer strategies has been greatly limited [131]. High levels of ROS and oxidative stress are significant markers of cancer. The adaptive metabolic reprogramming of cancer cells leads to increased ROS levels in cancer cells, and elevated ROS also regulates tumor metabolism and progression [80]. In recent years, more and more research studies have been conducted on the relationship between ROS and cancer. ROS is considered to be an important anticancer target due to the different roles of different levels of ROS in various stages of cancer development, and many studies have shown that therapeutic strategies targeting ROS are significantly effective in the treatment of various diseases, including human lung cancer, colorectal cancer, breast cancer, and glioblastoma [61,132,133,134]. Therefore, based on the effectiveness of excessive ROS for cancer treatment, new anticancer therapies targeting ROS generation can be regarded as a good solution to the current problems of cancer drug resistance and the low efficacy of traditional anticancer therapies.

### 3.1. Drug Therapies That Target ROS Generation

Currently, ROS-generation-based cancer treatment strategies have been widely used. Anticancer drugs are the traditional methods of cancer treatment. Some anticancer drugs are based on the inhibition of cancer cell proliferation and cell death caused by ROS generation, which has advantages over the resistance-based shortcomings of traditional drugs (such as 5-fluorouracil and cisplatin) [135]. On the other hand, many chemotherapeutic agents are able to alter redox homeostasis in cancer cells by increasing ROS generation to exert cancer therapeutic effects [136]. For example, the highly selective inhibitor NCT503 enhances the efficacy of temozolomide (a first-line chemotherapeutic agent for glioblastoma treatment) through ROS-mediated DNA damage and the Wnt/β-catenin pathway’s inhibition of key proteins in the de novo serine synthesis pathway, up-regulated in many cancers [134]. Moreira et al. demonstrated that celastrol, a natural pentyclic triterpenoid, exerted pro-oxidative activity based on the excessive accumulation of ROS to induce DSBs, apoptotic/necrotic cell death, and the inhibition of cell proliferation, therefore showing promise as a drug for the treatment of drug-resistant colon cancer [137]. In addition, urolithin B, a gastrointestinal final metabolite of polyphenols, inhibits the growth of colon cancer cells by remodeling the intestinal microbiome and tumor immune microenvironment [138]. Meanwhile, plumbagin, a natural compound whose anticancer principle is ROS generation, has been used in the treatment of various cancers, including oral cancer and breast cancer [114,139]. Selenium is considered to be a multi-target drug, and high-dose sodium selenite has increased ovarian cancer cell death by inducing ROS generation and GSH depletion to mediate iron death [140]. Therefore, drug therapies targeting ROS generation also play an important role in modern cancer treatment.

In addition to pharmacotherapy, ROS generation is also widely used in several novel anticancer therapies, such as photodynamic therapy (PDT), sonodynamic therapy (SDT), chemodynamic therapy (CDT), and the combination of multiple therapeutic therapies.

### 3.2. Novel Therapies Targeting ROS Generation

PDT is a new type of anticancer therapy in which ROS generated during treatment induces cell death through oxidative damage to cells (such as mitochondria, DNA, proteins, and cell membranes). Existing research studies show that PDT has a good effect on the treatment of cervical cancer, colon cancer, skin cancer, and lung cancer and that it has fewer adverse effects than surgery, chemotherapy, and radiation treatment [141,142]. PDT is a photochemical reaction. Its anticancer principle is that, in an environment containing molecular oxygen, photosensitizers entering tumor cells are activated by the light irradiation of a specific wavelength to stimulate the generation of toxic levels of reactive oxygen species and induce cell death [143]. In PDT therapy, photosensitizers smoothly reach specific tumor cells and are slowly eliminated as a key step of effective treatment. Effective delivery methods, such as ones that include nanoparticles (the key carriers of photosensitizers), have become indispensable means to improve the efficiency and specificity of PDT, such as bimetallic nanoparticles [144,145]. Therefore, the unique working principle of PDT makes it highly specific to cancer treatment.

Although PDT has the ability to accurately target ROS generation, it has certain limitations, such as limited light penetration depth, which is not ideal for the treatment of deep tumors [146]. SDT uses ultrasounds to penetrate deep into biological tissues and overcome penetration barriers for the treatment of deep tumors, showing good selectivity [147]. In addition, the combination of multiple therapeutic therapies is also an effective way to control cancer. The combination of PDT and chemotherapy can increase the toxicity of drugs to cells through photo-dependent ROS generation and thus exert increased synergistic antitumor effects [146,148]. Zhan et al. used nanopotentiators to reshape adenosine metabolism for SDT and CDT in combination with immunotherapy for deep tumors [149]. Moreover, Zhu et al. established a starvation-assisted, photothermal-thriving chemo/chemodynamic combined therapy to enhance CDT therapy by providing H_2_O_2_ [150]. Overall, it can be seen that the combination of multiple therapeutic therapies are also a development direction for modern cancer treatment.

### 3.3. Pro-Oxidative Anticancer Agents Targeting ROS Generation

**Figure 4:**
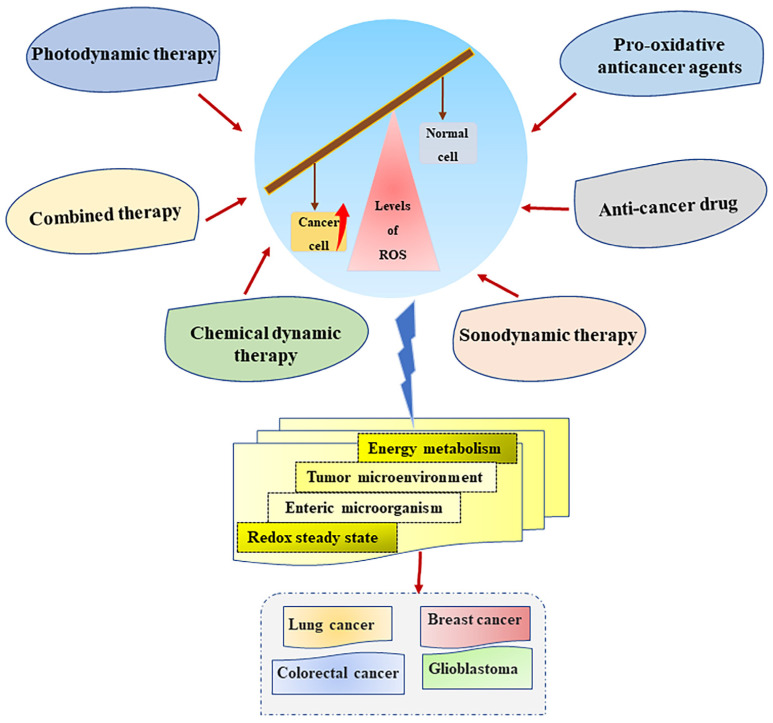
Cancer treatment strategies based on ROS generation. Multifarious cancer treatment strategies, such as anticancer drugs, photodynamic therapy, sonodynamic therapy, and the combination of multiple therapies can regulate metabolism, the tumor microenvironment, and the redox homeostasis of cancer cells by targeting ROS generation to exert a good anticancer effect. Abbreviations: ROS, reactive oxygen species.

Pro-oxidative anticancer agents (PAAs) targeting redox imbalance are effective for the treatment of cancer patients [151]. PAA is an effective means of directly targeting cellular ROS generation to inhibit tumor development and induce cell death. The metabolic pathways that distinguish cancer cells from normal cells place cancer cells in a state of oxidative stress, which makes them more susceptible to the influence of PAA, thus generating high levels of ROS. It was found that the key to the anticancer activity of Brevilin A was to induce ROS-dependent apoptosis [57,152]. There are already PAAs that selectively generate ROS in cancer cells. Various ROS-responsive drug delivery systems developed using ROS-responsive materials and connectors hold promising prospects for specific therapies targeting cancer [153]. In addition, a dietary curcumin mono-carbonyl analog designed by Liu et al. can selectively generate ROS in NCI-H460 cells of nude mice [154]. Therefore, the development of PAA with cancer-specific ROS generation can reduce the adverse effects caused by non-selective PAA and improve its safety, which is a very promising cancer treatment strategy. At present, targeting the generation of high levels of ROS is an effective strategy for cancer treatment. Specifically, the development and application of new specific anticancer therapy methods such as PDT, SDT, and multiple combination drug therapies make the treatment of various types of cancer more promising (Figure 4).

However, compared with contemporary conventional anticancer therapies and some other novel anticancer therapies, anticancer therapies targeting ROS generation within cancer cells have unique advantages but also certain limitations. Traditional surgery and chemotherapy drugs may have good efficacy in the early treatment of cancer, but the effectiveness of the treatment of malignant tumors is insufficient, and there are side effects and a high risk of recurrence. Cancer cells are resistant to treatments such as chemotherapy and radiotherapy and cannot be eradicated because of their properties such as rapid metabolic adaptation, high proliferation, and other resistance mechanisms. Cancer stem cells (CSC) exert resistance in radiotherapy and chemotherapy, which is the resistance mechanism of cancer treatment. For example, breast CSCs have traditional hyperthermia and radiation resistance [155]. Photothermal therapy (PTT) has been reported to sensitize CSCs to radiotherapy because of the ability of photothermal agents to affect ROS generation through local temperature control, and combination therapy (PDT/PTT) has shown high efficiency in the eradication of cancer stem cells [156]. However, this therapy has high requirements for the stability and accurate delivery of the photosensitizer. In addition, immune checkpoint blockade (ICB) therapy has been shown to be promising for cancer treatment. The principle is that immune checkpoint inhibitors block negative immune regulators and reactivate the antitumor immune system to increase the effect of cancer treatment. At present, it has been approved for the treatment of some advanced cancers. However, following its increasing application, clinical treatment has shown that this therapy has great drawbacks, such as non-specific targeting and serious tissue, organ, and systemic damage [157]. Fang et al. used topical hydrogel combined with ICB therapy to induce cancer cell death by providing continuous ROS and RNS and to minimize the systemic toxicity of ICB therapy [158]. However, different types and different stages of tumor development require effective delivery systems to deliver ROS to specific tumor sites. Therefore, there is an urgent need to develop effective and safe delivery systems to provide a basis for the universal application of this combination therapy. The challenge of targeting ROS generation-related therapies remains.

## 4. Discussion

Adaptive metabolic alterations lead to the abnormal accumulation of ROS in cancer cells, and cancer cells rely heavily on ROS levels to enhance tumorigenesis or induce apoptosis [80,159]. This review article systematically summarizes the dual roles of ROS accumulation in cancer cells in promoting and suppressing cancer, as well as the DNA damage and cancer cell apoptosis induced by toxic levels of ROS in cancer cells, providing basic information on how to find and identify effective anticancer treatments that excel in “targeting ROS generation” in cancer metabolic pathways.

In clinical practice, in order to cope with the highly invasive and metastatic nature of malignant tumors, it is often necessary to take chemotherapy drugs for a long time. However, the long-term and excessive use of chemotherapy drugs will not only increase the risk of harmful side effects and drug resistance of cancer cells, but also cause disrupt the tumor immune microenvironment and immune escape. Therefore, traditional cancer drugs do not have an advantage in the ongoing battle against cancer. At present, PDT, SDT, and CDT, which are commonly used in clinical practice, have certain advantages in traditional cancer treatment. However, the treatment of highly drug-resistant tumors is still a major problem, often requiring the combination of two or more treatments. Cancer drug resistance is a headache, which means that many chemotherapy drugs are not effective enough to kill cancer cells, and anticancer drugs targeting ROS generation can cause the apoptosis of drug-resistant cancer cells. Patchouli alcohol has been shown to have potential in the treatment of non-small cell lung cancer with or without vincristine resistance by inducing ROS-mediated DNA damage and promoting cell apoptosis [160]. However, anticancer therapies targeting ROS generation also have certain limitations, and only ROS levels that reach the toxicity threshold can play a role in killing tumor cells. However, due to tumor heterogeneity, high or low intracellular ROS levels are still not clearly defined, so the threshold for specific toxic levels of ROS is uncertain. To continuously generate high levels of ROS in as many cancer cells as possible, it is necessary to ensure the stable and continuous generation of ROS in the targeted sites, synthesize efficient and stable photosensitizers, and establish a precise delivery system for photosensitizers [161,162].

A variety of anticancer therapies can induce the generation of ROS to trigger the apoptosis of cancer cells; however, at the same time, the intrinsic detoxification mechanism of the body against ROS-mediated oxidative stress will also be activated. Autophagy can remove oxidative damage of organelles and biomolecules and induce the drug resistance of cancer cells, thus creating resistance to the cytotoxic effect of ROS [163]. The aggressive bone-malignant tumor osteosarcoma (OS) still has no significant effect on multimodal treatment. Since autophagy may play a role in maintaining the survival of osteosarcoma cells, Saini H et al. demonstrated that the use of autophagy inhibitors can disrupt multiple links of autophagy and make OS cells sensitive to drugs [164]. Therefore, autophagy regulation is an important factor that prevents toxic levels of ROS from promoting the apoptosis of cancer cells. In the future, part of our efforts will be devoted to the development of autophagy inhibitors to improve the anticancer effect of existing therapies by regulating autophagy.

The rapid metabolic adaptation of cancer cells occurs to meet the needs of endogenous growth, proliferation, and metastasis. The crosstalk between the complex pathways activated in cancer metabolic reprogramming has still not been fully elucidated, and the specific mechanism of metabolic changes regarding the generation and influence of high levels of ROS needs to be further elucidated. In addition, the specific mechanism of action of different ROS types in cancer cells in each stage of cancer development needs to be studied further, and the question of how to accurately characterize and locate each ROS type is also a problem to be solved. Traditional quantitative ROS detection techniques, such as chemical and immunological techniques, cannot meet the requirements of clinical detection standards. Therefore, the development of temporal- and spatial-specific ROS detection techniques to elucidate the complex biological mechanism of ROS-mediated redox in cancer is of great significance [165]. The solution to these scientific issues will promote the formulation of different cancer types and individualized anticancer therapy methods.

Due to the inherent heterogeneity of tumors, current drug treatments and therapies are unable to meet the increasingly severe changes with respect to cancer deterioration situations, and with the continuous adaptation of malignant tumors to the hypoxic microenvironment and the development of drug resistance through changes in material and energy metabolism, other obstacles have become increasingly prominent, posing great challenges to clinical cancer therapy. ROS are effective anticancer targets, and anticancer therapies targeting ROS are proven to be effective means of cancer treatment. However, among the various therapeutic methods used to induce cancer cell apoptosis through ROS generation, the effectiveness and specificity of such treatments still need to be improved. Therefore, it is necessary to ensure that the generation of ROS with therapeutic effects is selective and specific for cancer-type cells. This should be the goal when developing anticancer drugs that target ROS generation. Therefore, based on the important role of ROS levels in the regulation of cancer, the wide application of anticancer therapy methods targeting ROS generation is promising for cancer treatment.

## Figures and Tables

**Table 1:** Main exogenous factors of ROS generation.

Objective	Aim	Results	Reference
Air pollutants	To demonstrate that oxidative stress is the key mechanism by which secondary organic aerosols affect cell health.	To a large extent, cellular health depends on cellular ROS levels, and secondary organic aerosols exposure produce biological effects through oxidative stress.	[37]
	To investigate mechanism of oxidative stress and cytotoxicity induced by DEPs exposure in HBE.	Cells treated with DEPs showed high levels of ROS generation and oxidative DNA damage.	[60]
Anticancer drugs	To investigate whether ononin synergizes with paclitaxel to inhibit non-small-cell lung cancer progression and promote apoptosis in vitro and in vivo.	The combination of ononin and paclitaxel increased the expression of ROS generation and apoptotic markers and inhibited cell proliferation through the PI3K/Akt/mTOR signaling pathways.	[61]
	To investigate mechanism of action regarding redox-active quinone chelators.	Redox-active quinone chelators can be reduced by ETC and GSH while generating ROS. They can also generate ROS upon photoexcitation.	[62]
	To explore the anticancer mechanism of Brv-A.	Brv-A induces ROS generation; however, Brv-A regulates ROS generation through NOX2 and NOX3 proteins and the induction of ER-stress in MCF-7 BC cells.	[57]
	To investigate the antitumor mechanism of Agr.	Agr increased ROS, blocked Bcl-2 expression, and increased Caspase-3 and Bax expression to promote apoptosis in cancer cells.	[41]
	To investigate the effect of curcumin on the effect of irinotecan on CRC cells.	Curcumin enhances the killing effect of irinotecan on CRC cells by mediating increased ROS production and the activation of the ER stress pathway.	[63]
	To determine the effect and mechanism of action of curcumin analog WZ35 against human prostate cancer.	WZ35 exhibited strong antitumor potential against PC-3 cells by inducing ROS generation and subsequently inducing ER stress-dependent apoptosis and cell cycle arrest.	[64]
	To reveal the mechanism of WZ35-mediated ROS generation and amino acid metabolism regulation that inhibit gastric cancer cell metastasis.	WZ35 can deplete the GSH reserve by increasing ROS generation. The mechanism maintains the GSH consumption phenotype through the ROS-YAP-AXL-ALKBH5-GLS2 loop.	[65]
	To investigate the molecular mechanism of human BC cells apoptosis caused by SH003.	SH003 induced apoptosis in BC cells by increasing ROS generation and activating the ER stress signaling pathways.	[66]
Chemicals	To explore the key mechanism of methamphetamine in HCC.	ROS-mediated Ras up-regulation activates the MEK/ERK signaling pathway, which is key mechanism by which methamphetamine promotes HCC progress.	[67]
	To investigate the antitumor mechanism of organic arsenic compound Aa-Z2 on osteosarcoma by targeting cancer metabolism.	Aa-Z2 induces ROS accumulation by targeting PDK-1 to induce osteosarcoma apoptosis.	[42]
	To explore the mechanism of mitochondrial damage after exposure to toxic polypropylene nanoplastic.	Polypropylene nanoplastic stimulation leads to mitochondrial dysfunction and ROS generation and causes lung inflammation through the p38-mediated NF-κB pathway.	[68]
Radiation	To evaluate the combined action of EMFs and black carbon particles in the HL-60 promyelocytic cell line exposed to 2.45 GHz RF radiation.	The interaction between black carbon particles and RF leads to ROS generation and triggers oxidative stress to activate necrosis/apoptosis, leading to long-term cytotoxicity.	[69]
Heavy metals	To study the co-carcinogenic effects of UVB and arsenic on mouse epidermal cell line JB6 and its mechanism.	Arsenic enhances UVB-induced ROS generation and causes DNA damage and apoptosis in mouse skin cells.	[70]
	To investigate the ROS generation and related bio-effects of various metal-porphyrin complexes under ultrasonic exposure.	Zn(II) and Pd(II) porphyrins are the most effective in producing singlet oxygen and hydroxyl radicals, and the different patterns of ROS generation depend on the metal moiety.	[71]
	To investigate the role of ROS- associated autophagy in Cd-induced cell proliferation and the invasion of A549 cells.	Exposure to Cd (2 μM) significantly increased ROS accumulation, induced autophagy, and enhanced cell growth, migration, and invasion in A549 cells.	[72]
	To investigate the effects of low concentrations of Cd on the regulation of liver cancer cell proliferation, steatosis, and fibrogenic/ oncogenic signaling.	Exposure to Cd (1–10 nM) increases ROS production, cell proliferation, steatosis, and fibrogenic/oncogenic signaling by activating Notch and AKT/mTOR pathways.	[38]
Cigarette smoke	To assess the toxic effects of PAH and HEV light combination in human RPE cells.	Toxic synergistic interaction between IcdP and HEV light. This synergy translates into the disruption of the mitochondrial network, enhanced ROS accumulation, and apoptosis.	[73]
Cytokine	To investigate the role of IL-13 and 13(S)HpODE (endogenous product during IL-13 activation) in mediating apoptotic pathways in three different in vitro cellular models: A549 lung cancer, HCT116 colorectal cancer, and CCF52 GBM cells.	13(S)HpODE significantly reduces solid tumor growth through the activation of apoptosis. IL-13 and 13(S)HpODE participate in activating the p53-p21 signaling cascade via MAO-A-mediated ROS and ultimately induce apoptosis by inhibiting Bcl-2 and promoting Bax.	[59]
FRGs	To explore and verify the mechanism by which FRGs promotes the progression and invasion of colon adenocarcinoma.	FRGs improves tumor cell survival by activating the TGFB pathway, which can stimulate ROS generation, accelerate ECM decomposition, and promote tumor progression and invasion.	[58]
Mitochondria damage	To investigate the molecular mechanism of ouabain-induced ROS generation and apoptosis in human GBM cells.	Ouabain-induced GBM cells apoptosis increased ROS generation through the phosphorylation of p66Shc (mediated by the Src/Ras/ERK signaling pathways).	[54]
NOX	To investigate in GBM cell lines that cause TGF-β1 to drive metabolic reprogramming and aggressive cancer by enhancing NOX4 activity.	TGF-β1 up-regulated NOX4 expression accompanied by ROS through Smad-dependent signaling and then induced HIF-1α overexpression and metabolic reprogramming while promoting EMT (modulated by the PI3K/AKT/HIF-1α signaling pathways).	[74]
	To investigate the role of NOX complex proteins in the Cd-induced malignant transformation of prostate epithelial cells and the molecular mechanisms involved.	Chronic Cd exposure activated NOX1 complex proteins and generated ROS and ER stress, which led to defective autophagy.	[75]

Abbreviations: 13(S)HpODE, 13-(S)-hydroperoxyoctadecadienoic acid; Agr, Agrimol B; Bax, Bcl2-associated X protein; BC, breast cancer; Bcl-2, B-cell lymphoma 2; Brv-A, brevilin A; Cd, cadmium; CRC, colorectal cancer; DEPs, diesel exhaust particles; ECM, extracellular matrix; EMFs, electromagnetic fields; EMT, epithelial-mesenchymal transition; ER, endoplasmic reticulum; ETC, electron transfer chain; FRGs, ferroptosis-related genes; GBM, glioblastoma multiforme; GHz, gigahertz; GSH, glutathione; HBE, human bronchial epithelium; HCC, hepatocellular carcinoma; HEV, high-energy visible blue; HIF-1α, hypoxia-inducible factor-1α; IcdP, indeno [1,2, 3-cd]pyrene; IL-13, interleukin 13; MCF-7 BC, MAO-A, monoamine oxidase-A; MCF-7 breast carcinoma cells; NF-κB, nuclear transcription factor kappa B; NOX, NADPH oxidase; NOX1, NADPH oxidase 1; NOX2, NADPH oxidase 2; NOX3, NADPH oxidase 3; NOX4, NADPH oxidase 4; PAH, polycyclic aromatic hydrocarbons; PC-3, human prostate cancer cells; Pd, palladium; PDK-1, pyruvate dehydrogenase kinase 1; PI3K/Akt/mTOR, phosphatidylinositol 3-kinase (PI3K)/AKT/mammalian target of rapamycin (mTOR) pathway; Ras, renin–angiotensin system; RF, radio frequency; ROS, reactive oxygen species; RPE, human retinal pigment epithelium cells; SH003, a newly developed herbal medicine; TGFB, transforming growth factor beta; TGF-β1, transforming growth factor- β1; UVB, ultraviolet b; Zn, zinc.

## References

[1] Sung H., Ferlay J., Siegel R.L., Laversanne M., Soerjomataram I., Jemal A., Bray F. (2021). Global cancer statistics 2020: GLOBOCAN estimates of incidence and mortality worldwide for 36 cancers in 185 countries. CA Cancer J. Clin..

[2] Hernandez D., Cheng C.Y., Hernandez-Villafuerte K., Schlander M. (2022). Survival and comorbidities in lung cancer patients: Evidence from administrative claims data in Germany. Oncol. Res..

[3] Ezzati M., Riboli E. (2013). Behavioral and dietary risk factors for noncommunicable diseases. N. Engl. J. Med..

[4] Aune D., Chan D.S.M., Lau R., Vieira R., Greenwood D.C., Kampman E., Norat T. (2011). Dietary fibre, whole grains, and risk of colorectal cancer: Systematic review and dose-response meta-analysis of prospective studies. BMJ.

[5] Siegel R.L., Fedewa S.A., Anderson W.F., Miller K.D., Ma J., Rosenberg P.S., Jemal A. (2017). Colorectal cancer incidence patterns in the United States, 1974-2013. J. Natl. Cancer Inst..

[6] Araghi M., Soerjomataram I., Jenkins M., Brierley J., Morris E., Bray F., Arnold M. (2019). Global trends in colorectal cancer mortality: Projections to the year 2035. Int. J. Cancer.

[7] Lucky S.S., Soo K.C., Zhang Y. (2015). Nanoparticles in photodynamic therapy. Chem. Rev..

[8] Alfarouk K.O., Stock C.M., Taylor S., Walsh M., Muddathir A.K., Verduzco D., Bashir A.H., Mohammed O.Y., Elhassan G.O., Harguindey S. (2015). Resistance to cancer chemotherapy: Failure in drug response from ADME to P-gp. Cancer Cell Int..

[9] Bergamini C., Leoni I., Rizzardi N., Melli M., Galvani G., Coada C.A., Giovannini C., Monti E., Liparulo I., Valenti F. (2023). MiR-494 induces metabolic changes through G6pc targeting and modulates sorafenib response in hepatocellular carcinoma. J. Exp. Clin. Cancer Res..

[10] Chio I.I.C., Tuveson D.A. (2017). ROS in Cancer: The Burning Question. Trends Mol. Med..

[11] Qian S., Fang Y., Yao C., Wang Y., Zhang Z., Wang X., Gao J., Feng Y., Sun L., Zou R. (2022). The synergistic effects of PRDX5 and Nrf2 on lung cancer progression and drug resistance under oxidative stress in the zebrafish models. Oncol. Res..

[12] Ebata H., Loo T.M., Takahashi A. (2022). Telomere maintenance and the cGAS-STING pathway in cancer. Cells.

[13] Morin G.B. (1989). The human telomere terminal transferase enzyme is a ribonucleoprotein that synthesizes TTAGGG repeats. Cell.

[14] Cuadrado M., Martinez-Pastor B., Fernandez-Capetillo O. (2006). ATR activation in response to ionizing radiation: Still ATM territory. Cell Div..

[15] McKinnon P.J. (2012). ATM and the molecular pathogenesis of ataxia telangiectasia. Annu. Rev. Pathol..

[16] Huiting W., Dekker S.L., van der Lienden J.C.J., Mergener R., Musskopf M.K., Furtado G.V., Gerrits E., Coit D., Oghbaie M., Di Stefano L.H. (2022). Targeting DNA topoisomerases or checkpoint kinases results in an overload of chaperone systems, triggering aggregation of a metastable subproteome. eLife.

[17] Priya B., Ravi S., Kirubakaran S. (2023). Targeting ATM and ATR for cancer therapeutics: Inhibitors in clinic. Drug Discov. Today.

[18] Kavec M.J., Urbanova M., Makovicky P., Opattová A., Tomasova K., Kroupa M., Kostovcikova K., Siskova A., Navvabi N., Schneiderova M. (2022). Oxidative damage in sporadic colorectal cancer: Molecular mapping of base excision repair glycosylases MUTYH and hOGG1 in colorectal cancer patients. Int. J. Mol. Sci..

[19] Hoesel B., Schmid J.A. (2013). The complexity of NF-κB signaling in inflammation and cancer. Mol. Cancer.

[20] Vander Heiden M.G., DeBerardinis R.J. (2017). Understanding the intersections between metabolism and cancer biology. Cell.

[21] Pavlova N.N., Thompson C.B. (2016). The emerging hallmarks of cancer metabolism. Cell Metab..

[22] Pavlova N.N., Zhu J., Thompson C.B. (2022). The hallmarks of cancer metabolism: Still emerging. Cell Metab..

[23] Warburg O. (1956). On the origin of cancer cells. Science.

[24] Vander Heiden M.G., Cantley L.C., Thompson C.B. (2009). Understanding the Warburg effect: The metabolic requirements of cell proliferation. Science.

[25] Hanahan D., Weinberg R.A. (2011). Hallmarks of cancer: The next generation. Cell.

[26] Chang C.H., Qiu J., O’Sullivan D., Buck M.D., Noguchi T., Curtis J.D., Chen Q., Gindin M., Gubin M.M., van der Windt G.J. (2015). Metabolic competition in the tumor microenvironment is a driver of cancer progression. Cell.

[27] Shu P., Liang H., Zhang J., Lin Y., Chen W., Zhang D. (2023). Reactive oxygen species formation and its effect on CD4(+) T cell-mediated inflammation. Front. Immunol..

[28] Chen Y.J., Guo X., Liu M.L., Yu Y.Y., Cui Y.H., Shen X.Z., Liu T.S., Liang L. (2022). Interaction between glycolysis-cholesterol synthesis axis and tumor microenvironment reveal that gamma-glutamyl hydrolase suppresses glycolysis in colon cancer. Front. Immunol..

[29] Martínez-Reyes I., Chandel N.S. (2021). Cancer metabolism: Looking forward. Nat. Rev. Cancer.

[30] Pizzino G., Irrera N., Cucinotta M., Pallio G., Mannino F., Arcoraci V., Squadrito F., Altavilla D., Bitto A. (2017). Oxidative stress: Harms and benefits for human health. Oxid. Med. Cell Longev..

[31] Sinha B.K., Tokar E.J., Bortner C.D. (2022). Molecular mechanisms of cytotoxicity of NCX4040, the non-steroidal anti-inflammatory NO-donor, in human ovarian cancer cells. Int. J. Mol. Sci..

[32] Reuter S., Gupta S.C., Chaturvedi M.M., Aggarwal B.B. (2010). Oxidative stress, inflammation, and cancer: How are they linked?. Free Radic. Biol. Med..

[33] Sinha B.K., Tokar E.J., Li J., Bushel P.R. (2023). Gene expression profiling elucidates cellular responses to NCX4040 in human ovarian tumor cells: Implications in the mechanisms of action of NCX4040. Cancers.

[34] Popovici V., Musuc A.M., Matei E., Karampelas O., Ozon E.A., Cozaru G.C., Schröder V., Bucur L., Aricov L., Anastasescu M. (2022). ROS-induced DNA-damage and autophagy in oral squamous cell carcinoma by *Usnea barbata* oil extract-an in vitro study. Int. J. Mol. Sci..

[35] Shah M.A., Rogoff H.A. (2021). Implications of reactive oxygen species on cancer formation and its treatment. Semin. Oncol. Cancer.

[36] Valavanidis A., Fiotakis K., Vlachogianni T. (2008). Airborne particulate matter and human health: Toxicological assessment and importance of size and composition of particles for oxidative damage and carcinogenic mechanisms. J. Environ. Sci. Health Pt. C-Environ. Carcinog. Ecotoxicol. Rev..

[37] Liu F., Xu T., Ng N.L., Lu H. (2023). Linking cell health and reactive oxygen species from secondary organic aerosols exposure. Environ. Sci. Technol..

[38] Niture S., Gadi S., Lin M., Qi Q., Niture S.S., Moore J.T., Bodnar W., Fernando R.A., Levine K.E., Kumar D. (2023). Cadmium modulates steatosis, fibrosis, and oncogenic signaling in liver cancer cells by activating notch and AKT/mTOR pathways. Environ. Toxicol..

[39] Mitra D., Luo X., Morgan A., Wang J., Hoang M.P., Lo J., Guerrero C.R., Lennerz J.K., Mihm M.C., Wargo J.A. (2012). An ultraviolet-radiation-independent pathway to melanoma carcinogenesis in the red hair/fair skin background. Nature.

[40] D’Orazio J., Jarrett S., Amaro-Ortiz A., Scott T. (2013). UV radiation and the skin. Int. J. Mol. Sci..

[41] Xiang D., Yang W., Fang Z., Mao J., Yan Q., Li L., Tan J., Yu C., Qian J., Tang D. (2022). Agrimol B inhibits colon carcinoma progression by blocking mitochondrial function through the PGC-1α/NRF1/TFAM signaling pathway. Front. Oncol..

[42] Liu Y., She W., Li Y., Wang M., Liu Y., Ning B., Xu T., Huang T., Wei Y. (2023). Aa-Z2 triggers ROS-induced apoptosis of osteosarcoma by targeting PDK-1. J. Transl. Med..

[43] Gómez-Sierra T., Jiménez-Uribe A.P., Ortega-Lozano A.J., Ramírez-Magaña K.J., Pedraza-Chaverri J., Litwack G. (2023). Antioxidants affect endoplasmic reticulum stress-related diseases. Vitamins and Hormones: Antioxidants.

[44] Yang L., Li A., Wang Y., Zhang Y. (2023). Intratumoral microbiota: Roles in cancer initiation, development and therapeutic efficacy. Signal Transduct. Target. Ther..

[45] Scherz-Shouval R., Elazar Z. (2011). Regulation of autophagy by ROS: Physiology and pathology. Trends Biochem. Sci..

[46] Romesberg A., Van Houten B. (2022). Targeting mitochondrial function with chemoptogenetics. Biomedicines.

[47] Sylvester A.L., Zhang D.X., Ran S., Zinkevich N.S. (2022). Inhibiting NADPH oxidases to target vascular and other pathologies: An update on recent experimental and clinical studies. Biomolecules.

[48] Bánfi B., Clark R.A., Steger K., Krause K.H. (2003). Two novel proteins activate superoxide generation by the NADPH oxidase NOX1. J. Biol. Chem..

[49] Juhasz A., Markel S., Gaur S., Liu H., Lu J., Jiang G., Wu X., Antony S., Wu Y., Melillo G. (2017). NADPH oxidase 1 supports proliferation of colon cancer cells by modulating reactive oxygen species-dependent signal transduction. J. Biol. Chem..

[50] Chan J.S., Tan M.J., Sng M.K., Teo Z., Phua T., Choo C.C., Li L., Zhu P., Tan N.S. (2018). Cancer-associated fibroblasts enact field cancerization by promoting extratumoral oxidative stress. Cell Death Dis..

[51] Moldovan L., Moldovan N.I. (2004). Oxygen free radicals and redox biology of organelles. Histochem. Cell Biol..

[52] Wallace D.C. (2012). Mitochondria and cancer. Nat. Rev. Cancer.

[53] Denisenko T.V., Gorbunova A.S., Zhivotovsky B. (2019). Mitochondrial involvement in migration, invasion and metastasis. Front. Cell Dev. Biol..

[54] Yan X., Liang F., Li D., Zheng J. (2015). Ouabain elicits human glioblastoma cells apoptosis by generating reactive oxygen species in ERK-p66SHC-dependent pathway. Mol. Cell Biochem..

[55] Liu-Smith F., Dellinger R., Meyskens F.L. (2014). Updates of reactive oxygen species in melanoma etiology and progression. Arch. Biochem. Biophys..

[56] Zhang X., Li H., Liu C., Yuan X. (2022). Role of ROS-mediated autophagy in melanoma (Review). Mol. Med. Rep..

[57] Saleem M.Z., Nisar M.A., Alshwmi M., Din S.R.U., Gamallat Y., Khan M., Ma T. (2020). Brevilin A inhibits STAT3 signaling and induces ROS-dependent apoptosis, mitochondrial stress and endoplasmic reticulum stress in MCF-7 breast cancer cells. Onco Targets Ther..

[58] Baldi S., He Y., Ivanov I., Sun Y., Feng W., Refat M., Mohammed S.A.D., Adlat S., Tian Z., Wang Y. (2022). Novel characterization discoveries of ferroptosis-associated molecules in COAD microenvironment based TCGA data. Front. Mol. Biosci..

[59] Biswas P., Swaroop S., Dutta N., Arya A., Ghosh S., Dhabal S., Das P., Majumder C., Pal M., Bhattacharjee A. (2023). IL-13 and the hydroperoxy fatty acid 13(S)HpODE play crucial role in inducing an apoptotic pathway in cancer cells involving MAO-A/ROS/p53/p21 signaling axis. Free Radic. Biol. Med..

[60] Kwon M., Jung J., Park H.S., Kim N.H., Lee J., Park J., Kim Y., Shin S., Lee B.S., Cheong Y.H. (2023). Diesel exhaust particle exposure accelerates oxidative DNA damage and cytotoxicity in normal human bronchial epithelial cells through PD-L1. Environ. Pollut..

[61] Gong G., Ganesan K., Xiong Q., Zheng Y. (2022). Antitumor effects of ononin by modulation of apoptosis in non-small-cell lung cancer through inhibiting PI3K/Akt/mTOR pathway. Oxid. Med. Cell Longev..

[62] Polyakov N., Leshina T., Fedenok L., Slepneva I., Kirilyuk I., Furso J., Olchawa M., Sarna T., Elas M., Bilkis I. (2018). Redox-active quinone chelators: Properties, mechanisms of action, cell delivery, and cell toxicity. Antioxid. Redox Signal..

[63] Huang Y.F., Zhu D.J., Chen X.W., Chen Q.K., Luo Z.T., Liu C.C., Wang G.X., Zhang W.J., Liao N.Z. (2017). Curcumin enhances the effects of irinotecan on colorectal cancer cells through the generation of reactive oxygen species and activation of the endoplasmic reticulum stress pathway. Oncotarget.

[64] Zhang X., Chen M., Zou P., Kanchana K., Weng Q., Chen W., Zhong P., Ji J., Zhou H., He L. (2015). Curcumin analog WZ35 induced cell death via ROS-dependent ER stress and G2/M cell cycle arrest in human prostate cancer cells. BMC Cancer.

[65] Chen T., Chen J., Zeng T., Huang Q., Chen D., Chen H., Chen J., Zheng B., Wang M., Chen S. (2023). WZ35 inhibits gastric cancer cell metastasis by depleting glutathione to promote cellular metabolic remodeling. Cancer Lett..

[66] Lee S.Y., Kim T.H., Choi W.G., Chung Y.H., Ko S.G., Cheon C., Cho S.G. (2023). SH003 causes ER stress-mediated apoptosis of breast cancer cells via intracellular ROS production. Cancer Genom. Proteom..

[67] Si Z., Yang G., Wang X., Yu Z., Pang Q., Zhang S., Qian L., Ruan Y., Huang J., Yu L. (2023). An unconventional cancer-promoting function of methamphetamine in hepatocellular carcinoma. Life Sci. Alliance.

[68] Woo J.H., Seo H.J., Lee J.Y., Lee I., Jeon K., Kim B., Lee K. (2023). Polypropylene nanoplastic exposure leads to lung inflammation through p38-mediated NF-κB pathway due to mitochondrial damage. Part. Fibre Toxicol..

[69] Benavides R.A.S., Leiro-Vidal J.M., Rodriguez-Gonzalez J.A., Ares-Pena F.J., López-Martín E. (2023). The HL-60 human promyelocytic cell line constitutes an effective in vitro model for evaluating toxicity, oxidative stress and necrosis/apoptosis after exposure to black carbon particles and 2.45 GHz radio frequency. Sci. Total Environ..

[70] Yin Y., Meng F., Sui C., Jiang Y., Zhang L. (2019). Arsenic enhances cell death and DNA damage induced by ultraviolet B exposure in mouse epidermal cells through the production of reactive oxygen species. Clin. Exp. Dermatol..

[71] Giuntini F., Foglietta F., Marucco A.M., Troia A., Dezhkunov N.V., Pozzoli A., Durando G., Fenoglio I., Serpe L., Canaparo R. (2018). Insight into ultrasound-mediated reactive oxygen species generation by various metal-porphyrin complexes. Free Radic. Biol. Med..

[72] Lv W., Sui L., Yan X., Xie H., Jiang L., Geng C., Li Q., Yao X., Kong Y., Cao J. (2018). ROS-dependent Atg4 upregulation mediated autophagy plays an important role in Cd-induced proliferation and invasion in A549 cells. Chem. Biol. Interact..

[73] Zinflou C., Rochette P.J. (2019). Absorption of blue light by cigarette smoke components is highly toxic for retinal pigmented epithelial cells. Arch. Toxicol..

[74] Su X., Yang Y., Guo C., Zhang R., Sun S., Wang Y., Qiao Q., Fu Y., Pang Q. (2021). NOX4-derived ROS mediates TGF-*β*1-induced metabolic reprogramming during epithelial-mesenchymal transition through the PI3K/AKT/HIF-1*α* pathway in glioblastoma. Oxid. Med. Cell Longev..

[75] Tyagi A., Chandrasekaran B., Navin A.K., Shukla V., Baby B.V., Ankem M.K., Damodaran C. (2023). Molecular interplay between NOX1 and autophagy in cadmium-induced prostate carcinogenesis. Free Radic. Biol. Med..

[76] Sosa V., Moliné T., Somoza R., Paciucci R., Kondoh H., LLeonart M.E. (2013). Oxidative stress and cancer: An overview. Ageing Res. Rev..

[77] Zhu H., Ma H., Dong N., Wu M., Li D., Liu L., Shi Q., Ju X. (2023). 1,5-anhydroglucitol promotes pre-B acute lymphocytic leukemia progression by driving glycolysis and reactive oxygen species formation. BMC Cancer.

[78] Wang Q., Zhang Q., Zhang Z., Ji M., Du T., Jin J., Jiang J.D., Chen X., Hu H.Y. (2023). Characterization of chlorogenic acid as a two-photon fluorogenic probe that regulates glycolysis in tumor cells under hypoxia. J. Med. Chem..

[79] Circu M.L., Aw T.Y. (2010). Reactive oxygen species, cellular redox systems, and apoptosis. Free Radic. Biol. Med..

[80] Moloney J.N., Cotter T.G. (2018). ROS signalling in the biology of cancer. Semin. Cell Dev. Biol..

[81] Galadari S., Rahman A., Pallichankandy S., Thayyullathil F. (2017). Reactive oxygen species and cancer paradox: To promote or to suppress?. Free Radic. Biol. Med..

[82] Perillo B., Di Donato M., Pezone A., Di Zazzo E., Giovannelli P., Galasso G., Castoria G., Migliaccio A. (2020). ROS in cancer therapy: The bright side of the moon. Exp. Mol. Med..

[83] Barnes D.E., Lindahl T. (2004). Repair and genetic consequences of endogenous DNA base damage in mammalian cells. Annu. Rev. Genet..

[84] Burrows C.J., Muller J.G. (1998). Oxidative nucleobase modifications leading to strand scission. Chem. Rev..

[85] Li C., Xue Y., Ba X., Wang R. (2022). The role of 8-oxoG repair systems in tumorigenesis and cancer therapy. Cells.

[86] Boiteux S., Coste F., Castaing B. (2017). Repair of 8-oxo-7,8-dihydroguanine in prokaryotic and eukaryotic cells: Properties and biological roles of the Fpg and OGG1 DNA N-glycosylases. Free Radic. Biol. Med..

[87] Feng T., Zhao R., Sun F., Lu Q., Wang X., Hu J., Wang S., Gao L., Zhou Q., Xiong X. (2020). TXNDC9 regulates oxidative stress-induced androgen receptor signaling to promote prostate cancer progression. Oncogene.

[88] Tsai S.H., Huang P.H., Hsu Y.J., Peng Y.J., Lee C.H., Wang J.C., Chen J.W., Lin S.J. (2016). Inhibition of hypoxia inducible factor-1α attenuates abdominal aortic aneurysm progression through the down-regulation of matrix metalloproteinases. Sci. Rep..

[89] Zhang L., Cao Y., Guo X., Wang X., Han X., Kanwore K., Hong X., Zhou H., Gao D. (2023). Hypoxia-induced ROS aggravate tumor progression through HIF-1α-SERPINE1 signaling in glioblastoma. J. Zhejiang Univ.-SCI. B.

[90] Gao Y., Nan X., Shi X., Mu X., Liu B., Zhu H., Yao B., Liu X., Yang T., Hu Y. (2019). SREBP1 promotes the invasion of colorectal cancer accompanied upregulation of MMP7 expression and NF-κB pathway activation. BMC Cancer.

[91] Eller-Borges R., Rodrigues E.G., Teodoro A.C.S., Moraes M.S., Arruda D.C., Paschoalin T., Curcio M.F., da Costa P.E., Do Nascimento I.R., Calixto L.A. (2023). Bradykinin promotes murine melanoma cell migration and invasion through endogenous production of superoxide and nitric oxide. Nitric Oxide-Biol. Chem..

[92] Robinson A.J., Hopkins G.L., Rastogi N., Hodges M., Doyle M., Davies S., Hole P.S., Omidvar N., Darley R.L., Tonks A. (2020). Reactive oxygen species drive proliferation in acute myeloid leukemia via the glycolytic regulator PFKFB3. Cancer Res..

[93] Srinivas U.S., Tan B.W.Q., Vellayappan B.A., Jeyasekharan A.D. (2019). ROS and the DNA damage response in cancer. Redox Biol..

[94] Rowe L.A., Degtyareva N., Doetsch P.W. (2008). DNA damage-induced reactive oxygen species (ROS) stress response in *Saccharomyces cerevisiae*. Free Radic. Biol. Med..

[95] Ma F., Ma Y., Liu K., Gao J., Li S., Sun X., Li G. (2023). Resveratrol induces DNA damage-mediated cancer cell senescence through the DLC1-DYRK1A-EGFR axis. Food Funct..

[96] Yu T.T., Hu J., Li Q.R., Peng X.C., Xu H.Z., Han N., Li L.G., Yang X.X., Xu X., Yang Z.Y. (2023). Chlorin e6-induced photodynamic effect facilitates immunogenic cell death of lung cancer as a result of oxidative endoplasmic reticulum stress and DNA damage. Int. Immunopharmacol..

[97] Arjmand F., Yasir Khan H., Tabassum S. (2023). Progress of metal-based anticancer chemotherapeutic agents in last two decades and their comprehensive biological (DNA/RNA binding, cleavage and cytotoxicity activity) studies. Chem. Rec..

[98] Carmody R.J., Cotter T.G. (2001). Signalling apoptosis: A radical approach. Redox Rep..

[99] Wang Y., Guo S.H., Shang X.J., Yu L.S., Zhu J.W., Zhao A., Zhou Y.F., An G.H., Zhang Q., Ma B. (2018). Triptolide induces Sertoli cell apoptosis in mice via ROS/JNK-dependent activation of the mitochondrial pathway and inhibition of Nrf2-mediated antioxidant response. Acta Pharmacol. Sin..

[100] Kennedy N.J., Davis R.J. (2003). Role of JNK in tumor development. Cell Cycle.

[101] Wang X., Zhang S., Han K., Wang L., Liu X. (2022). Induction of apoptosis by matrine derivative ZS17 in human hepatocellular carcinoma BEL-7402 and HepG2 cells through ROS-JNK-P53 signalling pathway activation. Int. J. Mol. Sci..

[102] Xu H., Xu G., Xu Q., Xu C., Zhou X., Bai Y., Yin L., Ding Y., Wang W. (2022). MLN2238 exerts its anti-tumor effects via regulating ROS/JNK/mitochondrial signaling pathways in intrahepatic cholangiocarcinoma. Front. Pharmacol..

[103] Lv Y., Du Y., Li K., Ma X., Wang J., Du T., Ma Y., Teng Y., Tang W., Ma R. (2023). The FACT-targeted drug CBL0137 enhances the effects of rituximab to inhibit B-cell non-Hodgkin’s lymphoma tumor growth by promoting apoptosis and autophagy. Cell Commun. Signal..

[104] Groeger G., Quiney C., Cotter T.G. (2009). Hydrogen peroxide as a cell-survival signaling molecule. Antioxid. Redox Signal..

[105] Flores-Romero H., Hohorst L., John M., Albert M.C., King L.E., Beckmann L., Szabo T., Hertlein V., Luo X., Villunger A. (2022). BCL-2-family protein tBID can act as a BAX-like effector of apoptosis. EMBO J..

[106] Mehmood T., Maryam A., Tian X., Khan M., Ma T. (2017). Santamarine inhibits NF-кB and STAT3 activation and induces apoptosis in HepG2 liver cancer cells via oxidative stress. J. Cancer.

[107] Su J., Yan Y., Qu J., Xue X., Liu Z., Cai H. (2017). Emodin induces apoptosis of lung cancer cells through ER stress and the TRIB3/NF-κB pathway. Oncol. Rep..

[108] Dai X., Zhang J., Guo G., Cai Y., Cui R., Yin C., Liu W., Vinothkumar R., Zhang T., Liang G. (2018). A mono-carbonyl analog of curcumin induces apoptosis in drug-resistant EGFR-mutant lung cancer through the generation of oxidative stress and mitochondrial dysfunction. Cancer Manag. Res..

[109] Geraghty P., Wallace A., D’Armiento J.M. (2011). Induction of the unfolded protein response by cigarette smoke is primarily an activating transcription factor 4-C/EBP homologous protein mediated process. Int. J. Chron. Obstruct. Pulmon. Dis..

[110] Malhotra J.D., Miao H., Zhang K., Wolfson A., Pennathur S., Pipe S.W., Kaufman R.J. (2008). Antioxidants reduce endoplasmic reticulum stress and improve protein secretion. Proc. Natl. Acad. Sci. USA.

[111] Piao M.J., Han X., Kang K.A., Fernando P.D.S.M., Herath H.M.U.L., Hyun J.W. (2022). The endoplasmic reticulum stress response mediates shikonin-induced apoptosis of 5-fluorouracil-resistant colorectal cancer cells. Biomol. Ther..

[112] Chen W., Zou P., Zhao Z., Weng Q., Chen X., Ying S., Ye Q., Wang Z., Ji J., Liang G. (2016). Selective killing of gastric cancer cells by a small molecule via targeting TrxR1 and ROS-mediated ER stress activation. Oncotarget.

[113] Lin C.L., Yu C.I., Lee T.H., Chuang J.M., Han K.F., Lin C.S., Huang W.P., Chen J.Y., Chen C.Y., Lin M.Y. (2023). Plumbagin induces the apoptosis of drug-resistant oral cancer in vitro and in vivo through ROS-mediated endoplasmic reticulum stress and mitochondrial dysfunction. Phytomedicine.

[114] Pan G., O’Rourke K., Chinnaiyan A.M., Gentz R., Ebner R., Ni J., Dixit V.M. (1997). The receptor for the cytotoxic ligand TRAIL. Science.

[115] Huang R., Chen H., Liang J., Li Y., Yang J., Luo C., Tang Y., Ding Y., Liu X., Yuan Q. (2021). Dual role of reactive oxygen species and their application in cancer therapy. J. Cancer.

[116] Tsuzuki T., Egashira A., Igarashi H., Iwakuma T., Nakatsuru Y., Tominaga Y., Kawate H., Nakao K., Nakamura K., Ide F. (2001). Spontaneous tumorigenesis in mice defective in the MTH1 gene encoding 8-oxo-dGTPase. Proc. Natl. Acad. Sci. USA.

[117] Kakehashi A., Ishii N., Okuno T., Fujioka M., Gi M., Wanibuchi H. (2017). Enhanced susceptibility of Ogg1 mutant mice to multiorgan carcinogenesis. Int. J. Mol. Sci..

[118] Hamanaka R.B., Chandel N.S. (2010). Mitochondrial reactive oxygen species regulate cellular signaling and dictate biological outcomes. Trends Biochem. Sci..

[119] Chen W.M., Chiang J.C., Shang Z., Palchik G., Newman C., Zhang Y., Davis A.J., Lee H., Chen B.P. (2023). DNA-PKcs and ATM modulate mitochondrial ADP-ATP exchange as an oxidative stress checkpoint mechanism. EMBO J..

[120] Luo M., Wicha M.S. (2019). Targeting cancer stem cell redox metabolism to enhance therapy responses. Semin. Radiat. Oncol..

[121] Hayes J.D., Dinkova-Kostova A.T., Tew K.D. (2020). Oxidative stress in cancer. Cancer Cell.

[122] DeNicola G.M., Karreth F.A., Humpton T.J., Gopinathan A., Wei C., Frese K., Mangal D., Yu K.H., Yeo C.J., Calhoun E.S. (2011). Oncogene-induced Nrf2 transcription promotes ROS detoxification and tumorigenesis. Nature.

[123] Arslanbaeva L.R., Santoro M.M. (2020). Adaptive redox homeostasis in cutaneous melanoma. Redox Biol..

[124] Zimta A.A., Cenariu D., Irimie A., Magdo L., Nabavi S.M., Atanasov A.G., Berindan-Neagoe I. (2019). The role of Nrf2 activity in cancer development and progression. Cancers.

[125] Robertson H., Dinkova-Kostova A.T., Hayes J.D. (2020). NRF2 and the ambiguous consequences of its activation during initiation and the subsequent stages of tumourigenesis. Cancers.

[126] Jain A., Lamark T., Sjøttem E., Larsen K.B., Awuh J.A., Øvervatn A., McMahon M., Hayes J.D., Johansen T. (2010). p62/SQSTM1 is a target gene for transcription factor NRF2 and creates a positive feedback loop by inducing antioxidant response element-driven gene transcription. J. Biol. Chem..

[127] Wu S., Lu H., Bai Y. (2019). Nrf2 in cancers: A double-edged sword. Cancer Med..

[128] Wilson W.R., Hay M.P. (2011). Targeting hypoxia in cancer therapy. Nat. Rev. Cancer.

[129] Sun B., Yu L., Xu C., Li Y.M., Zhao Y.R., Cao M.M., Yang L.Y. (2021). NAD(P)HX epimerase downregulation promotes tumor progression through ROS/HIF-1α signaling in hepatocellular carcinoma. Cancer Sci..

[130] Willson J.A., Arienti S., Sadiku P., Reyes L., Coelho P., Morrison T., Rinaldi G., Dockrell D.H., Whyte M.K.B., Walmsley S.R. (2022). Neutrophil HIF-1α stabilization is augmented by mitochondrial ROS produced via the glycerol 3-phosphate shuttle. Blood.

[131] Boakye D., Jansen L., Halama N., Chang-Claude J., Hoffmeister M., Brenner H. (2021). Early discontinuation and dose reduction of adjuvant chemotherapy in stage III colon cancer patients. Ther. Adv. Med. Oncol..

[132] Zhang Z., Shen C., Zhou F., Zhang Y. (2023). Shikonin potentiates therapeutic efficacy of oxaliplatin through reactive oxygen species-mediated intrinsic apoptosis and endoplasmic reticulum stress in oxaliplatin-resistant colorectal cancer cells. Drug Dev. Res..

[133] Truong Hoang Q., Huynh K.A., Nguyen Cao T.G., Kang J.H., Dang X.N., Ravichandran V., Kang H.C., Lee M., Kim J.E., Ko Y.T. (2023). Piezocatalytic 2D WS_2_ nanosheets for ultrasound-triggered and mitochondria-targeted piezodynamic cancer therapy synergized with energy metabolism-targeted chemotherapy. Adv. Mater..

[134] Jin L., Kiang K.M., Cheng S.Y., Leung G.K. (2022). Pharmacological inhibition of serine synthesis enhances temozolomide efficacy by decreasing O^6^-methylguanine DNA methyltransferase (MGMT) expression and reactive oxygen species (ROS)-mediated DNA damage in glioblastoma. Lab. Investig..

[135] Atashi F., Vahed N., Emamverdizadeh P., Fattahi S., Paya L. (2021). Drug resistance against 5-fluorouracil and cisplatin in the treatment of head and neck squamous cell carcinoma: A systematic review. J. Dent. Res. Dent. Clin. Dent. Prospect..

[136] Nakamura H., Takada K. (2021). Reactive oxygen species in cancer: Current findings and future directions. Cancer Sci..

[137] Moreira H., Szyjka A., Paliszkiewicz K., Barg E. (2019). Prooxidative activity of celastrol induces apoptosis, DNA damage, and cell cycle arrest in drug-resistant human colon cancer cells. Oxid. Med. Cell Longev..

[138] Wang L., Chen J., Chen Q., Song H., Wang Z., Xing W., Jin S., Song X., Yang H., Zhao W. (2023). The gut microbiota metabolite urolithin B prevents colorectal carcinogenesis by remodeling microbiota and PD-L1/HLA-B. Oxid. Med. Cell Longev..

[139] Kawiak A., Domachowska A., Lojkowska E. (2019). Plumbagin increases paclitaxel-induced cell death and overcomes paclitaxel resistance in breast cancer cells through ERK-mediated apoptosis induction. J. Nat. Prod..

[140] Choi J.A., Lee E.H., Cho H., Kim J.H. (2023). High-dose selenium induces ferroptotic cell death in ovarian cancer. Int. J. Mol. Sci..

[141] Gawel A.M., Singh R., Debinski W. (2022). Metal-based nanostructured therapeutic strategies for glioblastoma treatment—An update. Biomedicines.

[142] Yokoi K., Yasuda Y., Kanbe A., Imura T., Aoki S. (2023). Development of wireless power-transmission-based photodynamic therapy for the induction of cell death in cancer cells by cyclometalated iridium(III) complexes. Molecules.

[143] Nkune N.W., Kruger C.A., Abrahamse H. (2022). Synthesis of a novel nanobioconjugate for targeted photodynamic therapy of colon cancer enhanced with cannabidiol. Oncotarget.

[144] Crous A., Abrahamse H. (2020). Effective gold nanoparticle-antibody-mediated drug delivery for photodynamic therapy of lung cancer stem cells. Int. J. Mol. Sci..

[145] Li G., Zhang W., Luo N., Xue Z., Hu Q., Zeng W., Xu J. (2021). Bimetallic nanocrystals: Structure, controllable synthesis and applications in catalysis, energy and sensing. Nanomaterials.

[146] Jiang W., Liang M., Lei Q., Li G., Wu S. (2023). The current status of photodynamic therapy in cancer treatment. Cancers.

[147] Zhang L., Yi H., Song J., Huang J., Yang K., Tan B., Wang D., Yang N., Wang Z., Li X. (2019). Mitochondria-targeted and ultrasound-activated nanodroplets for enhanced deep-penetration sonodynamic cancer therapy. ACS Appl. Mater. Interfaces.

[148] Greco G., Ulfo L., Turrini E., Marconi A., Costantini P.E., Marforio T.D., Mattioli E.J., Di Giosia M., Danielli A., Fimognari C. (2023). Light-enhanced cytotoxicity of doxorubicin by photoactivation. Cells.

[149] Zhan M., Wang F., Liu Y., Zhou J., Zhao W., Lu L., Li J., He X. (2023). Dual-cascade activatable nanopotentiators reshaping adenosine metabolism for sono-chemodynamic-immunotherapy of deep tumors. Adv. Sci..

[150] Zhu B., Zhang M., Chen Q., Li Z., Chen S., Zhu J. (2023). Starvation-assisted and photothermal-thriving combined chemo/chemodynamic cancer therapy with PT/MR bimodal imaging. Biomater. Sci..

[151] Chen Y., Li J., Zhao Z. (2021). Redox control in acute lymphoblastic leukemia: From physiology to pathology and therapeutic opportunities. Cells.

[152] Khan M., Maryam A., Saleem M.Z., Shakir H.A., Qazi J.I., Li Y., Ma T. (2020). Brevilin A induces ROS-dependent apoptosis and suppresses STAT3 activation by direct binding in human lung cancer cells. J. Cancer.

[153] Saravanakumar G., Kim J., Kim W.J. (2017). Reactive-oxygen-species-responsive drug delivery systems: Promises and challenges. Adv. Sci..

[154] Liu X., Cui H., Li M., Chai Z., Wang H., Jin X., Dai F., Liu Y., Zhou B. (2023). Tumor killing by a dietary curcumin mono-carbonyl analog that works as a selective ROS generator via TrxR inhibition. Eur. J. Med. Chem..

[155] Burke A.R., Singh R.N., Carroll D.L., Wood J.C., D’Agostino R.B., Ajayan P.M., Torti F.M., Torti S.V. (2012). The resistance of breast cancer stem cells to conventional hyperthermia and their sensitivity to nanoparticle-mediated photothermal therapy. Biomaterials.

[156] Najafabad B.K., Attaran N., Mahmoudi M., Sazgarnia A. (2023). Effect of photothermal and photodynamic therapy with cobalt ferrite superparamagnetic nanoparticles loaded with LCG and PpIX on cancer stem cells in MDA-MB-231 and A375 cell lines. Photodiagnosis Photodyn. Ther..

[157] Marin-Acevedo J.A., Chirila R.M., Dronca R.S. (2019). Immune checkpoint inhibitor toxicities. Mayo Clin. Proc..

[158] Fang T., Cao X., Shen B., Chen Z., Chen G. (2023). Injectable cold atmospheric plasma-activated immunotherapeutic hydrogel for enhanced cancer treatment. Biomaterials.

[159] Aggarwal V., Tuli H.S., Varol A., Thakral F., Yerer M.B., Sak K., Varol M., Jain A., Khan M.A., Sethi G. (2019). Role of reactive oxygen species in cancer progression: Molecular mechanisms and recent advancements. Biomolecules.

[160] Liang C.Y., Chang K.F., Huang Y.C., Huang X.F., Sheu G.T., Kuo C.F., Hsiao C.Y., Tsai N.M. (2023). Patchouli alcohol induces G(0)/G(1) cell cycle arrest and apoptosis in vincristine-resistant non-small cell lung cancer through ROS-mediated DNA damage. Thorac. Cancer.

[161] Zhang T., Yang X., Ou X., Lee M.M.S., Zhang J., Xu C., Yu X., Gong P., Lam J.W.Y., Zhang P. (2023). Tailoring the amphiphilic structure of zwitterionic AIE photosensitizers to boost antitumor immunity. Adv. Mater..

[162] Ma X., Zhou W., Zhang R., Zhang C., Yan J., Feng J., Rosenholm J.M., Shi T., Shen X., Zhang H. (2023). Minimally invasive injection of biomimetic Nano@Microgel for in situ ovarian cancer treatment through enhanced photodynamic reactions and photothermal combined therapy. Mater. Today Bio.

[163] Aniogo E.C., George B.P., Abrahamse H. (2021). Molecular effectors of photodynamic therapy-mediated resistance to cancer cells. Int. J. Mol. Sci..

[164] Saini H., Sharma H., Mukherjee S., Chowdhury S., Chowdhury R. (2021). Verteporfin disrupts multiple steps of autophagy and regulates p53 to sensitize osteosarcoma cells. Cancer Cell Int..

[165] Yang H., Villani R.M., Wang H., Simpson M.J., Roberts M.S., Tang M., Liang X. (2018). The role of cellular reactive oxygen species in cancer chemotherapy. J. Exp. Clin. Cancer Res..

